# Cardanol and Eugenol Sourced Sustainable Non-halogen Flame Retardants for Enhanced Stability of Renewable Polybenzoxazines

**DOI:** 10.3389/fchem.2020.00711

**Published:** 2020-09-30

**Authors:** Divambal Appavoo, Nagarjuna Amarnath, Bimlesh Lochab

**Affiliations:** Materials Chemistry Laboratory, Department of Chemistry, School of Natural Sciences, Shiv Nadar University, Greater Noida, India

**Keywords:** cardanol, eugenol, phosphazene, flame retardant, halogen-free, polybenzoxazine, reactive flame retardant

## Abstract

Olefin bonds participate in co-reaction with the benzoxazine functionality of the monomer and are one of the strategies used to affect the crosslink density of a polybenzoxazine network. In general, the double bond incorporation in starting material is usually catalyzed by expensive, rare earth metals affecting the sustainability of the reaction. The natural abundance of feedstocks with inherent double bonds may be a powerful platform for the development of novel greener structures, with potential applications in polymers. Here, we report the design, synthesis, and characterization of a biobased non-halogen flame retardant, consisting of naturally occurring phenols, eugenol (E), and cardanol (C). The presence of a covalently linked phosphazene (P) core allowed the synthesis of hexa-functional flame retardant molecules, abbreviated as EP and CP. The chemical structures of the synthesized EP and CP were confirmed by Fourier transform infrared (FTIR), nuclear magnetic resonance (^1^H, ^13^C, ^31^P NMR), and single crystal XRD (only in the case of EP). Their polymerization with cardanol sourced tri-oxazine benzoxazine monomer, C-trisapm, was followed by FTIR, NMR, and DSC studies. The thermal stability and flame retardant properties of the hybrid phosphazene-benzoxazine copolymers was determined by thermogravimetry analysis (TGA), limiting oxygen index (LOI), vertical burning, and smoke density analyses. SEM images of the char residues of the polymers with or without the addition of reactive phosphazene molecules confirmed the intumescent flame retarding mechanism. Current work highlights the utility of sustainable origin non-halogen flame retardant (FR) molecules and their utility in polybenzoxazine chemistry.

## Introduction

Polybenzoxazine (PBz) is an upcoming class of phenolic polymer that is obtained from the thermal ring-opening polymerization (ROP) reaction of benzoxazine monomers with or without the addition of a catalyst/initiator. Unlike most traditional phenol-formaldehyde resins, the formation of PBzs eliminates the release of any harmful byproducts. PBzs exhibit interesting properties such as high mechanical strength and thermal stability, appreciable chemical and water resistance, near-zero shrinkage during polymerization, low dielectric properties, and surface free energy even lower than PTFE, making them an important class of high-performance thermoset resins. The reagents generally required for the synthesis of benzoxazine monomer are phenol, amine, formaldehyde, and recently, formaldehyde-free benzoxazine was also reported (Tavernier et al., 2020). The vast abundance and structural variability of both the major constituents, i.e., phenol, and amine, gives the PBz framework tremendous flexibility in terms of its molecular design (Ishida and Froimowicz, 2017). Many polymers are subject to limitations because they use raw materials with a petroleum origin, meaning there are issues with sustainability, and many resources including phenols may become dispensable soon.

**Figure 1:**
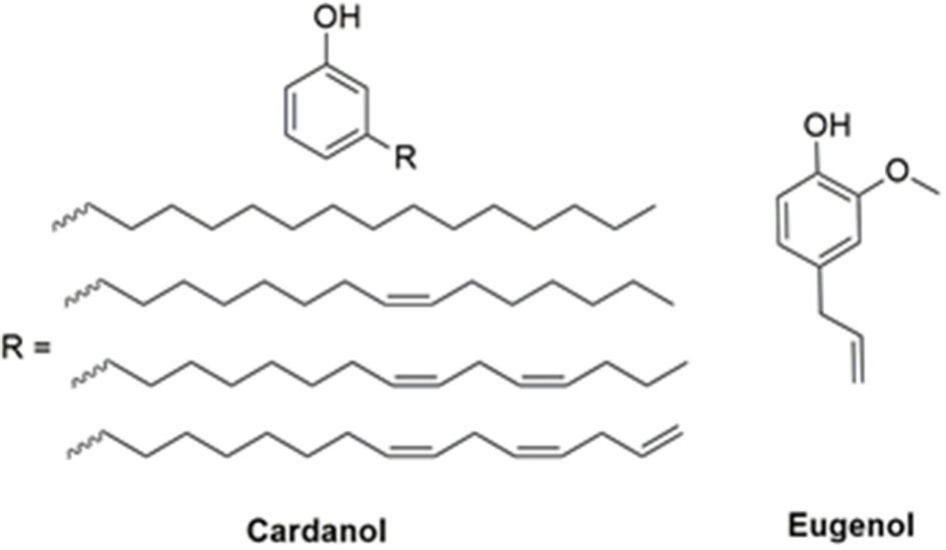
Structures of renewable phenols.

In this respect, the natural abundance of agro-origin sourced phenols, especially cardanol (Calò et al., 2007; Lochab et al., 2010, 2012; Shukla et al., 2015; Amarnath et al., 2018a; Monisha et al., 2018b) and eugenol (Thirukumaran et al., 2014a; Dumas et al., 2015; Amarnath et al., 2018b) hold great potential as substitutes for petro-based phenols in benzoxazine chemistry. Cardanol is derived from the mesocarp of a cashew nut, while eugenol is obtained largely through extraction from cloves and other plants (Lligadas et al., 2014; Lochab et al., 2014). Isolation of both these phenols is relatively simple and cost-effective. This makes them viable options with various potential applications at an industrial scale. Cardanol possesses a C-15 alkylene chain at the *meta*- position to the phenolic-OH with a varying degree of unsaturation (Tyman, 1973), while eugenol has an *ortho*-methoxy group and a propylene chain at the *para*- position to the phenolic-OH, as shown in Figure 1.

Owing to the structural differences of cardanol and eugenol, the resultant benzoxazines are expected to exhibit different sets of properties. The synthesis of cardanol and eugenol derived benzoxazines have been reported extensively in the literature. Depending on the functionality of the amine used, the resultant benzoxazine monomer showed mono- (Attanasi et al., 2012; Thirukumaran et al., 2014a, 2016a) bis- (Attanasi et al., 2012; Lligadas et al., 2014; Thirukumaran et al., 2014b; Dumas et al., 2015; Amarnath et al., 2019), tris- (Shukla et al., 2015), tetra- (Shukla et al., 2015), hexa- (Amarnath et al., 2018a), and octa-oxazine (Periyasamy et al., 2015) functionality. Similarly, for phenols, petroleum based amines are also being substituted by bio-based amines, such as furfurylamine, stearylamine, aminolysed poly(ethylene terephthalate), and isomannide diamine (ima) toward the synthesis of fully-sustainable benzoxazine monomers (Wang et al., 2012; Froidevaux et al., 2016; Sharma et al., 2016; Thirukumaran et al., 2016b; Amarnath et al., 2019). The double bonds in alkylene chain of cardanol are reported to undergo “autoxidation” reaction with air-oxygen (Xia et al., 2011; Ma et al., 2017). The mechanism involves a complex free-radical mechanism that forms a self-crosslinked film (John and Pillai, 1993; Honzícek, 2019). The various degrees of unsaturation in the side chain of cardanol mediate oxidative crosslinking reactions to form polymeric films and thus applications in surface coatings. Even UV irradiation, along with aerobic oxidative polymerization, has led to fast curable technology, expanding the prospects of different applications. Furthermore, the unsaturated C=C double bonds in eugenol sourced Bzs are reported to be involved in crosslinking reactions with benzoxazine ROP reaction (Amarnath et al., 2018b). Therefore, it is anticipated that double bonds are susceptible toward self-crosslinking or crosslinking with other monomers or intermediates formed during polymerization reactions.

In general, renewable origin Bz monomers, especially mono-oxazines, and those based on *o*-/*p*-substituted phenols containing non-crosslinking groups usually showed a low crosslink density which affects their flame resistance behavior. The approaches available are either increasing the oxazine functionality, which is governed by a higher functionality amine source, or by introducing functionalities that participate in multiple polymerization mechanisms. The scarcity of multifunctional amine structures means there is a need to explore alternative methodologies to enhance thermal stability. Consequently, naturally occurring phenols and bio-based amines have opened up new avenues to explore PBz as a green and sustainable polymer with the possibility for molecular flexibility for a variety of applications (Monisha et al., 2018a; Lyu and Ishida, 2019). However, the flame retardant property of renewable PBzs, and improvement in char yield, indicates that there is a need to explore greener facile methodologies. Although limited, this research has attracted the attention of many researchers (Zúñiga et al., 2013; Amarnath et al., 2018a).

Incorporation of silicon, phosphorus, and boron in PBz became an area of interest for boosting flame retardancy of PBz (Lin et al., 2006; Spontón et al., 2009; Cadiz et al., 2011; Ling and Gu, 2011; Zúñiga et al., 2013; Huang et al., 2015; Yan et al., 2016), but the incorporation of heteroatoms covalently into the benzoxazine monomer usually requires multiple synthetic steps, which is a major drawback for industrial-scale production and utility. The physical blending of reactive monomers containing such elements is a faster and better approach, which overcomes the usual leaching issues. Our group has recently reported the enhanced flame retardant properties of cardanol PBz using hexa-benzoxazine monomer with phosphazene core as an additive and the performance was attributed to the phosphazene ring in the structure (Amarnath et al., 2018a). Phosphazene is considered as an effective and safe substitute for halogen based flame retardants, which were banned due to their association with health and environmental concerns (Schartel et al., 2002; Bourbigot et al., 2004; Schartel, 2010; Shaw, 2010). Studies have reported that it is possible to incorporate flame retardancy into polybenzoxazine without the involvement of phosphorus, using raw materials containing high aromatic content (Zeng et al., 2020), however, further exploration is required to achieve the desired properties. The phosphorus-nitrogen synergism in phosphazene provides excellent flame retardant characteristics as they inhibit ignition and promote char formation without releasing any toxic gases. The potential molecule used to introduce a phosphazene core in many molecules is hexachlorotriphosphazene, N_3_P_3_Cl_6_, as it possesses six labile chlorine atoms, which provide an opportunity for structural modification (Allcock, 2003).

In this work, we are reporting an easy one-step synthetic route of hexacardanolphosphazene (CP) and hexaeugenolphosphazene (EP), which are explored as a reactive additive for benzoxazine monomer. We have prepared the reactive blends of CP/EP with cardanol based tris-benzoxazine monomer (C-trisapm) in different ratios to understand its effect in augmenting flame retardancy of resultant PBz. Along with the ROP, crosslinking of the unsaturated chains are also expected in an inter- and intramolecular fashion in the phosphazene and C-trisapm blends. The monomers, EP, CP, C-trisapm, were structurally characterized by nuclear magnetic resonance (^1^H, ^13^C, and ^31^P NMR), Fourier transform infrared (FTIR), single crystal X-ray diffraction (XRD), and mass spectrometry. Differential scanning calorimetry (DSC) and thermogravimetric analysis (TGA) were performed to analyze the polymerization temperature of the blends and thermal stability of copolymers, respectively. ^1^H NMR kinetic studies and non-isothermal FTIR studies were performed to understand the polymerization reaction in the blends. The flame retardant properties were determined by limiting oxygen index (LOI), vertical burning tests, and smoke density analysis. Scanning electron microscopy (SEM) and digital images of the char residue of homo- and co-polymers were used to understand the morphology and mode of the flame retardant (FR) mechanism in polymers.

## Experiment

### Materials

Cardanol was obtained from Satya Cashew Chemicals Pvt. Ltd. (India), sodium borohydride (10–40 mesh, 98%), eugenol (98%), and phosphonitrilic chloride trimer (99%) were purchased from Sigma Aldrich, paraformaldehyde from Fisher scientific, chloroform from Finar, anhydrous sodium sulfate and potassium carbonate from Chemlabs, and pararosaniline hydrochloride from CDH. All solvents used were AR grade and purified by standard procedures. CP^5^ and C-trisapm^24^ were prepared following the literature procedures.

#### Synthesis of Hexaeugenolcyclotriphosphazene (EP)

To a 250 mL two-neck round-bottom flask containing a mixtured of anhydrous acetone and anhydrous acetonitrile (3:1, 200 mL) under nitrogen atmosphere, activated K_2_CO_3_ (19.7 g, 143 mmol) was added. Eugenol (18.8 g, 1.15 mmol) was dissolved in acetone and added to the above mixture, followed by the addition of N_3_P_3_Cl_6_ (5.0 g, 14.3 mmol). The mixture was heated room temperature to 80°C and after 18 h of reaction, and the mixture was allowed to cool to room temperature and the solvent was evaporated. The residue was dissolved in ethyl acetate and the organic layer was washed with water, followed by 5% NaOH and water until the aqueous phase was neutral. The organic phase was dried over anhydrous sodium sulfate and the solvent was evaporated to give a light yellow liquid. Purification by column chromatography using 10% ethyl acetate in hexane gave a white solid of EP (12.0 g, 73%). ^1^H NMR (CDCl_3_, 400 MHz, ppm): 6.92–6.89 (d, ^3^*J*_H, H_ = 8, 6H; Ar*H*), 6.60 (s, 6H; Ar*H*), 6.48–6.46 (d, ^3^*J*_H, H_ = 8, 6H; Ar*H*), 5.94–5.84 (m, 6H; CH_2_=C*H*-CH_2_), 5.08–5.03 (m, 12H; C*H*_2_=C*H*-CH_2_), 3.64 (s, 18H; OC*H*_3_), 3.27 (d, ^3^*J*_H, H_ = 4, 12H; CH_2_=CH-C*H*_2_). ^13^C NMR (CDCl_3_, 100 MHz, ppm): 151.04 (Ar*C*), 139.10 (Ar*C*), 137.55 (CH_2_=*C*H-CH_2_), 136.68 (Ar*C*), 121.98 (Ar*C*), 120.37 (*C*H_2_=CH-CH_2_), 115.89 (Ar*C*), 113.02 (Ar*C*), 56.05 (O*C*H_3_), 40.15 (CH_2_=CH-*C*H_2_). ^31^P NMR (CDCl_3_, 162 MHz, ppm): 9.25. FTIR-ATR (diamond crystal/cm^−1^): 3,078, 2,982, 2,906, 2,829, 1,642, 1,601, 1,507, 1,177, 1,119, 950 cm^−1^. ESI(MS(+): [M+1]^+^ = 1114.39 (Calcd. 1113.39).

#### Synthesis of Hexacardanolcyclotriphosphazene (CP)

In a 500 mL two-neck round bottom flask, pre-dried cardanol (34.8 g, 115 mmol) was added with dried acetonitrile (120 mL), followed by activated K_2_CO_3_ (19.7 g, 143 mmol). A solution of N_3_P_3_Cl_6_ (5.0 g, 14 mmol) in dry acetonitrile (15 mL) was added to the cardanol solution. The mixture was heated to 85°C and stirred for 36 h. At the end of the reaction, the mixture was allowed to cool to room temperature and filtered. The residue was washed with acetonitrile to remove unreacted cardanol. Ethyl acetate was then added to dissolve the residue, washed with distilled water, 5% NaOH solution, and then again with distilled water until the aqueous phase was neutral. After drying the organic phase over anhydrous Na_2_SO_4_, the solvent was evaporated to give a dark brown liquid. Purification by column chromatography using 10% ethyl acetate in hexane eluent gave CP as a brown transparent liquid (19.1 g, 68%). ^1^H NMR (CDCl_3_, 400 MHz, ppm): 7.05-7.01 (t, ^3^*J*_H, H_ = 8, 6H; Ar*H*), 6.91–6.89 (d, ^3^*J*_H, H_ = 8, 6H; Ar*H*), 6.87 (s, 6H; Ar*H*), 6.74–6.72 (d, ^3^*J*_H, H_ = 8, 6H; Ar*H*), 5.87–5.77 (m, 2H, Alkene*H*), 5.47–5.30 (m, 26H, Alkene*H*), 5.07–4.97 (m, 4H, Alkene*H*), 2.83–2.76 (m, 14H, Alkyl*H*), 2.48–2.44 (t, ^3^*J*_H, H_ = 4,12H, ArC*H*_2_), 2.05–1.99 (m, 28H, Alkyl*H*), 1.5–1.25 (m, 114H, Alkyl*H*), 0.93–0.87 (m, 19H, Alkyl*H*). ^13^C NMR (CDCl_3_, 100 MHz, ppm): 150.91, 144.58, 136.91, 130.48, 130.05, 129.92, 129.41, 129.07, 128.27, 127.70, 126.94, 124.86, 121.05, 118.20, 114.83, 35.87, 31.93, 31.65, 31.36, 29.93, 29.88, 29.55, 29.50, 29.41, 29.13, 27.37, 25.78, 25.71, 22.93, 22.80, 14.25, 13.94. ^31^P NMR (CDCl_3_, 162 MHz, ppm): 8.24. FTIR-ATR (diamond crystal/cm^−1^): 3,008, 2,923, 2,855, 1,604, 1,583, 1,485, 1,446, 1,205, 1,142, 972.

### Characterization

Proton (^1^H), carbon (^13^C), and phosphorus (^31^P) NMR were used to verify the structures of the products with a Bruker AV400 NMR spectrometer at 400 MHz proton frequency and the corresponding carbon and phosphorus frequencies at room temperature in deuterated solvents with internal references tetramethylsilane and H_3_PO_4_. Signals were averaged from 16 transients for ^1^H and ^31^P NMR, and 256 transients for ^13^C NMR to yield spectra with sufficient signal-to-noise ratio. FTIR spectra were recorded on a Nicolet iS5 spectrometer equipped with attenuated total reflectance (iD5-ATR) accessory, in the range of 4,000–400 cm^−1^. Mass spectrometry (MS) analysis was carried out using Agilent HRMS Q-ToF 6540 Series equipped with ESI mode. The polymerization behavior of monomers was evaluated using differential scanning calorimetry DSC-3, Star System, Mettler Toledo. For DSC scans, samples (3 ± 2 mg) were enclosed in hermetic aluminum pans and heated from 30 to 350°C at 10°C/min under a constant flow rate of nitrogen at 50 mL/min. Prior to the experiments, the instrument was calibrated for temperature and enthalpy using standard indium and zinc. Thermal equilibrium was regained within 1 min of sample insertion, and the exothermic reaction was considered complete when the recorder signal leveled off to the baseline. TGA measurements of cured monomers were performed with a Perkin Elmer Diamond STG-DTA in the temperature range 30 to 800°C and a heating rate of 10°C/min in the air at a flow rate of 50 mL/min. The LOI of polymers was calculated from char yield obtained in the TGA data using Krevelen and Hoftyzer equation (Krevelen and Hoftyzer, 1976).

$$\begin{array}{l} LOI=17.5+0.4\;\times \;Char\;yield \end{array}$$

The “Smoke Density instrument” (according to ASTM international standards- Designation: D2843-16) was used as a means of measuring the relative amounts of smoke released during the burning or decomposition of the polymer samples.

$$\begin{array}{l} Smoke\;density\;rating=\frac{Area\;under\;the\;curve}{Total\;area}\times \;100 \end{array}$$

The vertical burning tests (UL-94) were conducted according to ASTM D 3801 standard. The surface morphology of samples was studied using a Scanning Electron Microscope (SEM) (ZEISS, EVO-MA10) under an acceleration voltage of 20 kV. The samples were coated with a thin layer of gold before testing to prevent electric discharge.

The swelling behavior of the polymer samples was determined by immersing around 25 mg each in various solvents at 30°C for different time intervals. The swelling ratio was calculated using the following equation

$$\begin{array}{l} Swelling\;ratio=\frac{m2-m1}{m1} \end{array}$$

Where, *m2*: Mass of the sample after swelling in the respective solvent for 96 h.

*m1*: Mass of the initial dry sample.

### NMR Kinetic Study and Calculation of Conversion of EP and CP to Other Species

The kinetics of the conversion of EP and CP to the new compounds was studied by ^1^H NMR spectroscopy. A test tube containing 200 mg of EP/CP was heated at 200°C under a nitrogen atmosphere, to prevent oxidation of double bonds to carbonyl functionalities. The trapped air was not degassed and remained in the test-tube to permit mild conditions for air-induced polymerization of double bonds and allowing ease of analysis of generated species. Aliquots were taken out at different time intervals and the content was suspended in CDCl_3_ and the test tube was re-purged with nitrogen. As the heating time increased, the solubility of the samples was found to decrease substantially, hence a longer suspension time (of up to 24 h) was allowed, to maximize solubility. The samples were filtered through PTFE filter (0.2 micron), and ^1^H NMR of the filtrate was recorded. The conversion of EP and CP to other respective product(s) was calculated by integrating the signals corresponding to the internal standard i.e., benzyl protons of the compounds (3.27 ppm for EP and 2.46 ppm for CP) (see Equation below). For EP and CP, the ratio of conversion was calculated from the integration of the well-defined newly formed resolvable signal at 3.32 and 2.55 ppm, respectively.

$$\begin{array}{l} Ratio\;of\;conversion=\frac{I_{XP}}{I_{XP^{\prime }}} \end{array}$$

where I_XP_ and ${\text{I}}_{\text{X}{\text{P}}^{\text{′}}}$ are the integrations of the signals corresponding to the newly formed and the original compound, respectively.

### Polymerization Study of the Blends

Three different weight ratios of CP or EP, C-trisapm of 3:1, 1:1, and 1:3, were prepared by mixing EP/CP and C-trisapm in THF. After evaporating the solvent under vacuum at 40°C for 2 h, the obtained homogeneous mixture was heated at 50, 100, 120, 160, 180, 200, 230, and 250°C for 1 h at each temperature. The blends are abbreviated as CP_x_T_y_ or EP_x_T_y_ where x and y are the weight ratios in the blends, and T is represented for C-trisapm. Conversion of weight ratio 3:1, 1:1, and 1:3 to molar ratio was calculated for both the blends and molar composition is found to be 3.4:1, 1.1:1, 1:2.6 for EP:C-trisapm and 2.0:1, 0.7:1, and 1:4.6 for CP:C-trisapm, respectively.

## Results and Discussion

### Synthesis and Characterization of CP, EP, and C-trisapm

**Scheme 1:**
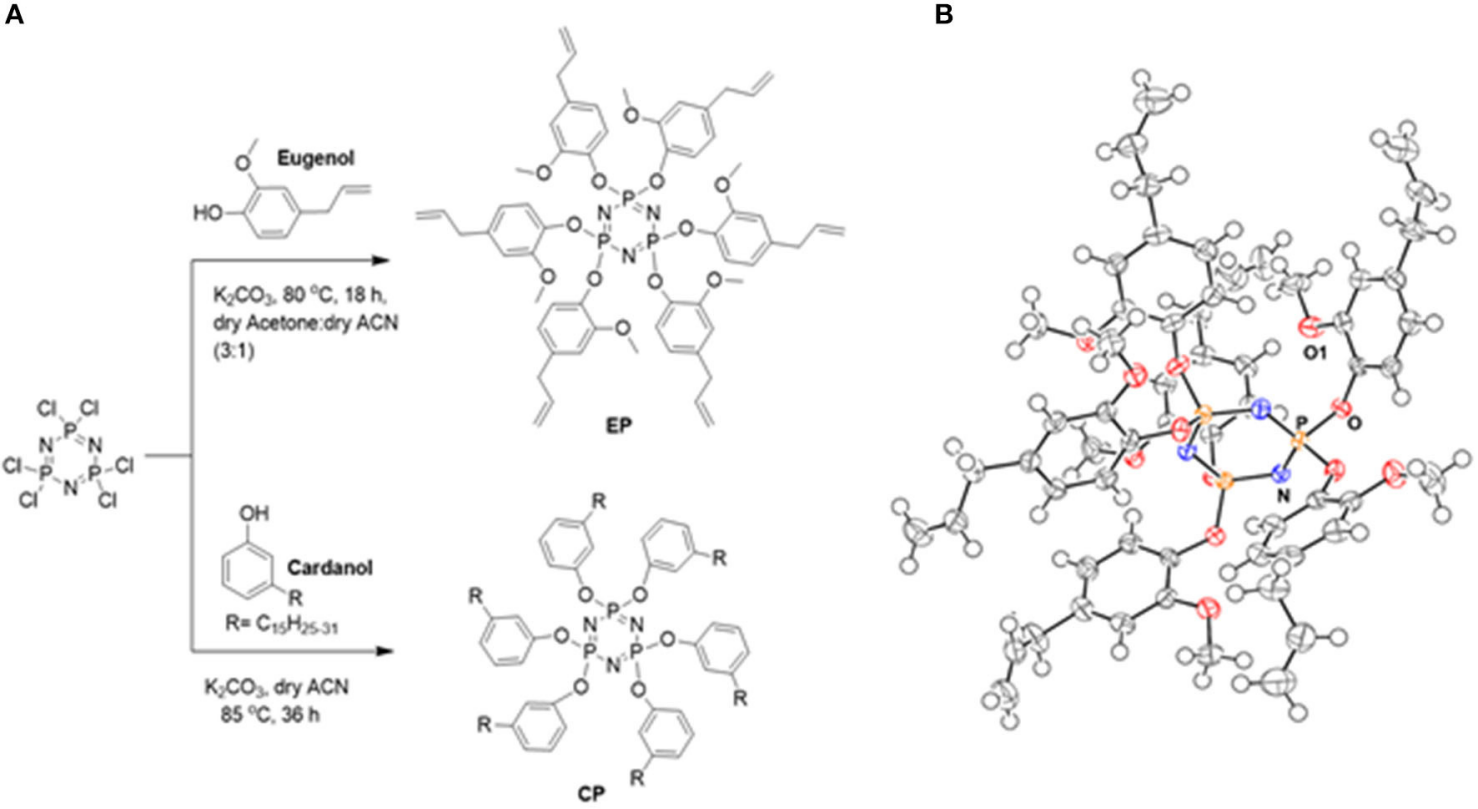
**(A)** Synthesis of EP and CP. **(B)** ORTEP diagram of EP.

Scheme 1 shows the method of preparation of CP and EP monomer. The reaction proceeded by a nucleophilic substitution reaction of the labile chlorine groups on the phosphazene ring with phenoxide ions sourced from cardanol (C) and eugenol (E). Acetonitrile was selectively used as a solvent in the case of cardanol, as it preferentially dissolves any unreacted cardanol while the product remains insoluble in the solvent. This allowed an easy separation of the CP monomer. In the case of eugenol reaction, the addition of acetone in acetonitrile was found to improve the yield. The earlier reported synthesis of EP utilized a sodium metal as a base, which is highly reactive, combustible, and corrosive. In the earlier reported synthesis of EP reaction proceeded in two-steps (Kireev et al., 2008), while the technique used in the current work involved a single-step and occurred in milder and safer conditions. Bz monomer, C-trisapm, were synthesized by the solventless reaction of cardanol and triarylamine (tris-apm) and characterized (details on synthesis and characterization are presented in the supporting information). The successful synthesis and purity of the synthesized monomers were confirmed by ^1^H, ^13^C, and ^31^P NMR, FTIR, and MS (for C-trisapm and EP). Monocrystals of EP, suitable for X-ray crystallography were obtained by slow evaporation of the solvent, confirming its star-shaped structure (as shown in Scheme 1B).

**Figure 2:**
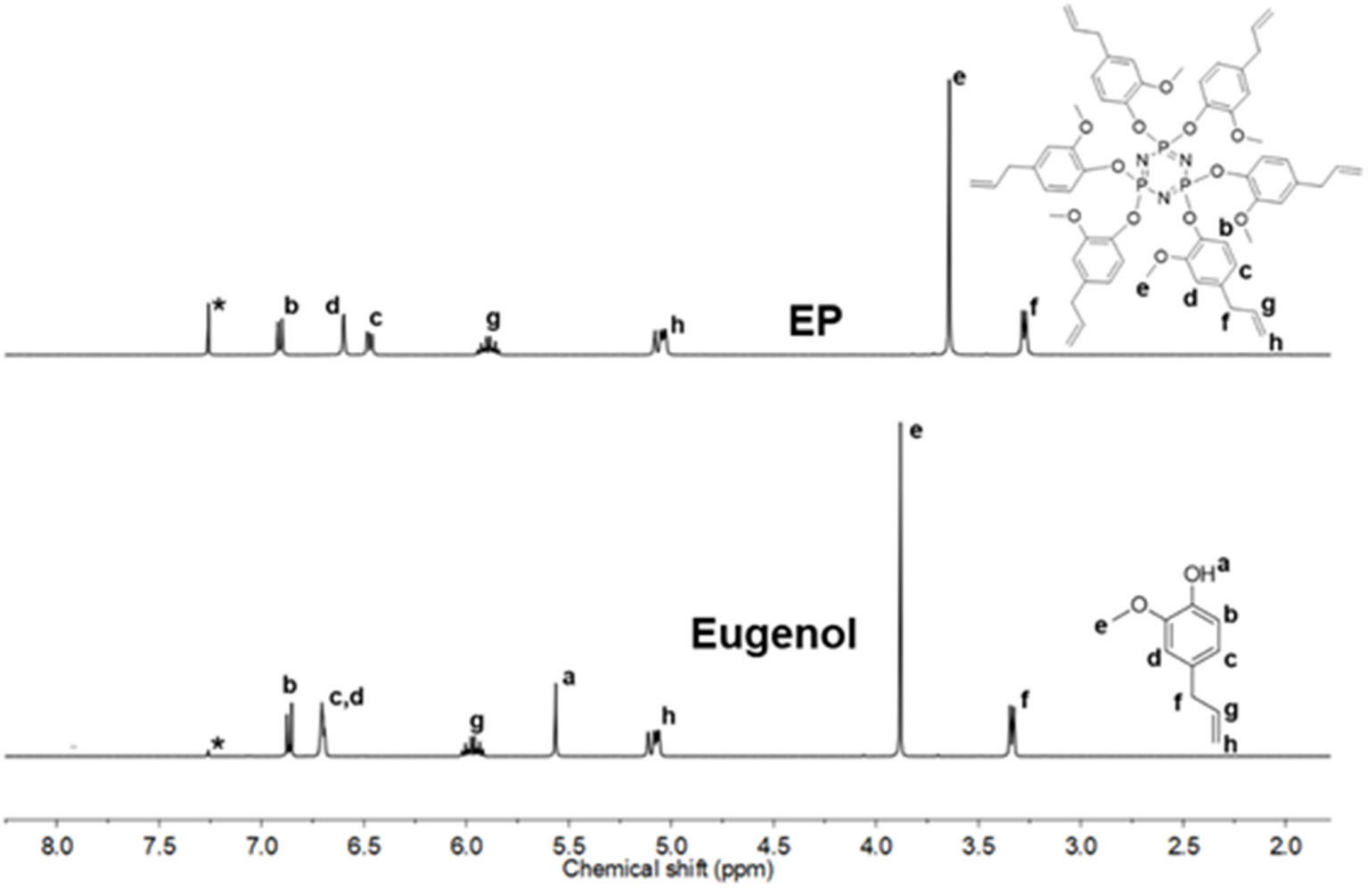
^1^H NMR spectra stack of EP and eugenol (Solvent*: CDCl_3_).

**Figure 3:**
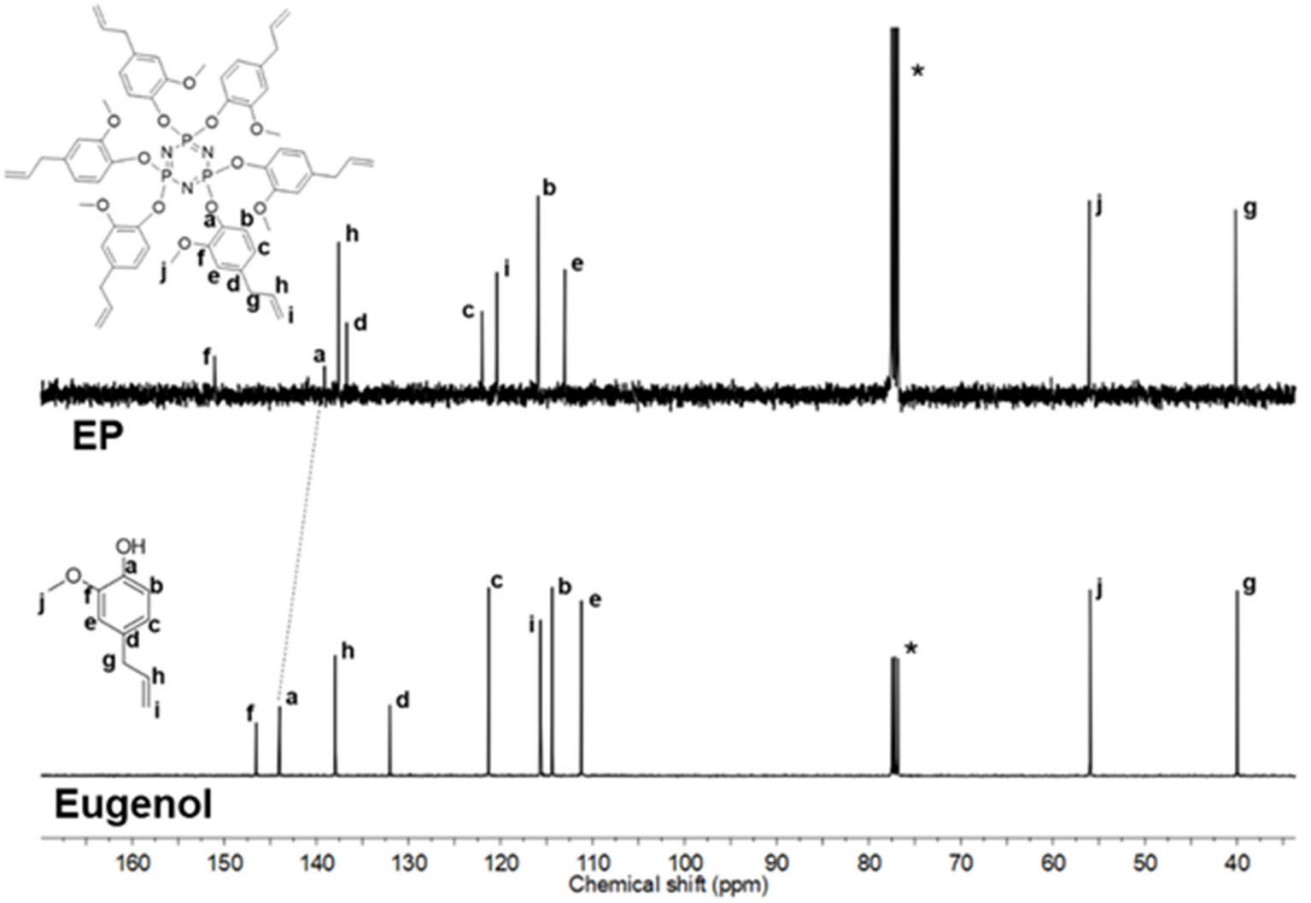
^13^C NMR spectra of EP and eugenol (Solvent: CDCl_3_).

**Figure 4:**
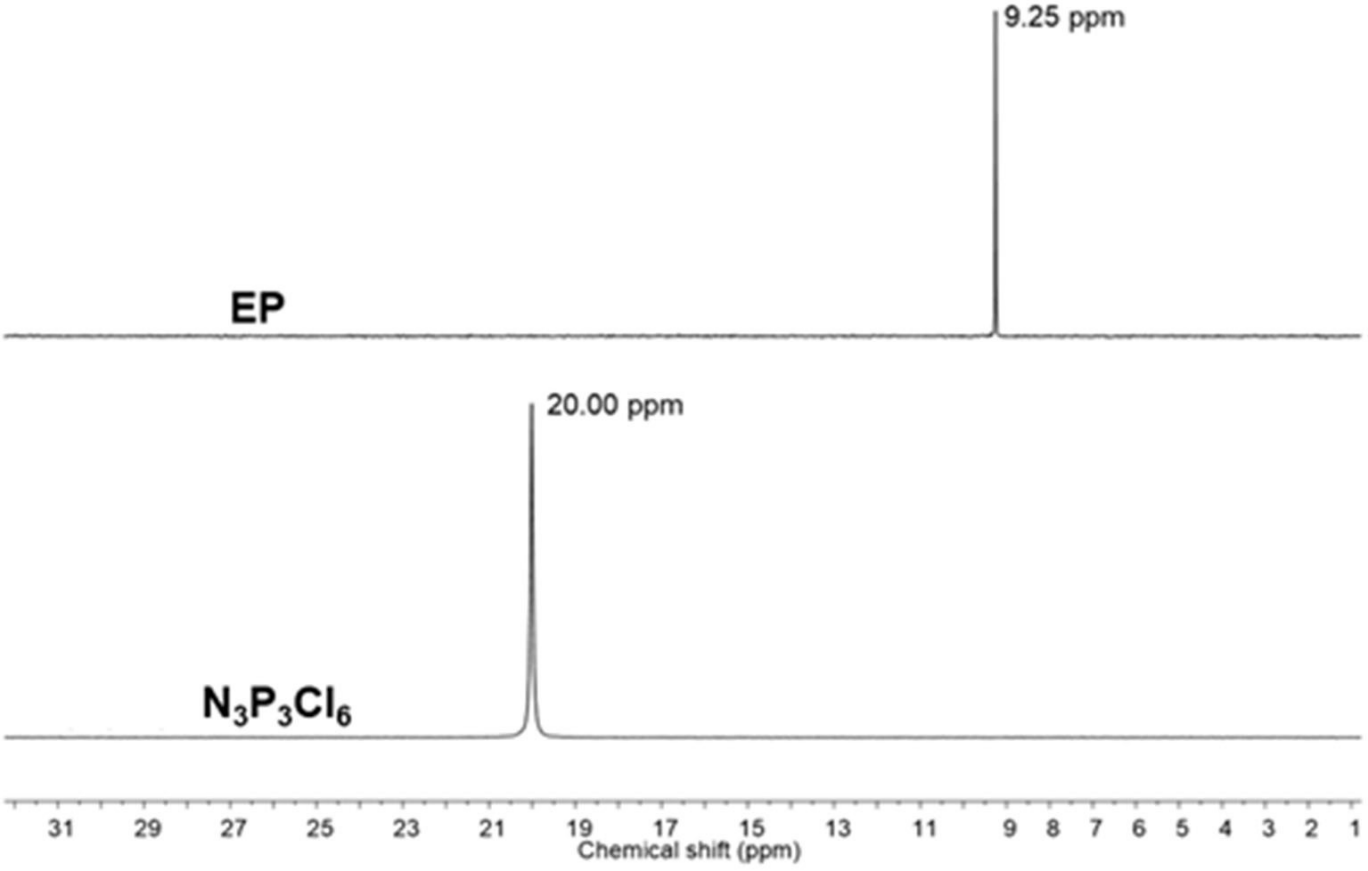
^31^P NMR spectra of EP and N_3_P_3_Cl_6_ (Solvent: CDCl_3_).

^1^H NMR spectra of EP and CP were compared to their respective phenol (Figure 2 and Supplementary Figure 1). The signal at 5.56 ppm in the eugenol spectrum, corresponds to the phenolic OH peak, which disappeared in the spectrum of EP (in the region 5.2–5.8 ppm), indicating the absence of eugenol as an impurity in EP. The cardanol-OH signal overlaps with the proton signals of the alkylene chain. Both EP and CP, the proton signal patterns in the aromatic region changed compared to the starting phenol spectra, supporting the reaction at the phenolic position that affected the environment of the aromatic protons. A general upfield shift of the proton signals was observed in CP and EP, attributed to the shielding effect of the phosphazene ring (Amarnath et al., 2018a). ^13^C NMR spectra of EP and CP are displayed in Figure 3 and Supplementary Figure 2, along with that of eugenol and cardanol. The chemical shifts of the aromatic C signals showed a shift while the aliphatic carbons remain unchanged. The most significant shift in both cases is that of the *C*-OH C signal (from 144 to 139 ppm in EP and from 155 to 150 ppm in CP) resulting from the increased shielding around the aromatic C of *C*-O-P. ^31^P NMR is a straightforward and sensitive technique for the characterization of phosphorus, containing compounds since a slight change in the phosphorus environment is reflected in the NMR spectrum. Figure 4 and Supplementary Figure 3 show the stacked ^31^P NMR of synthesized molecules and N_3_P_3_Cl_6_. A clear chemical shift of the phosphorus signal is observed from 20.00 ppm in N_3_P_3_Cl_6_ to 9.25 ppm in EP, and 8.24 ppm in CP. The presence of only one signal in the ^31^P NMR spectra of EP and CP supports the formation of only one compound, confirming all three phosphorus in the same environment and formation of hexa-phenol functional phosphazene monomer. Complete substitution of all Cl atoms in N_3_P_3_Cl_6_ by phenolic groups was confirmed.

**Figure 5:**
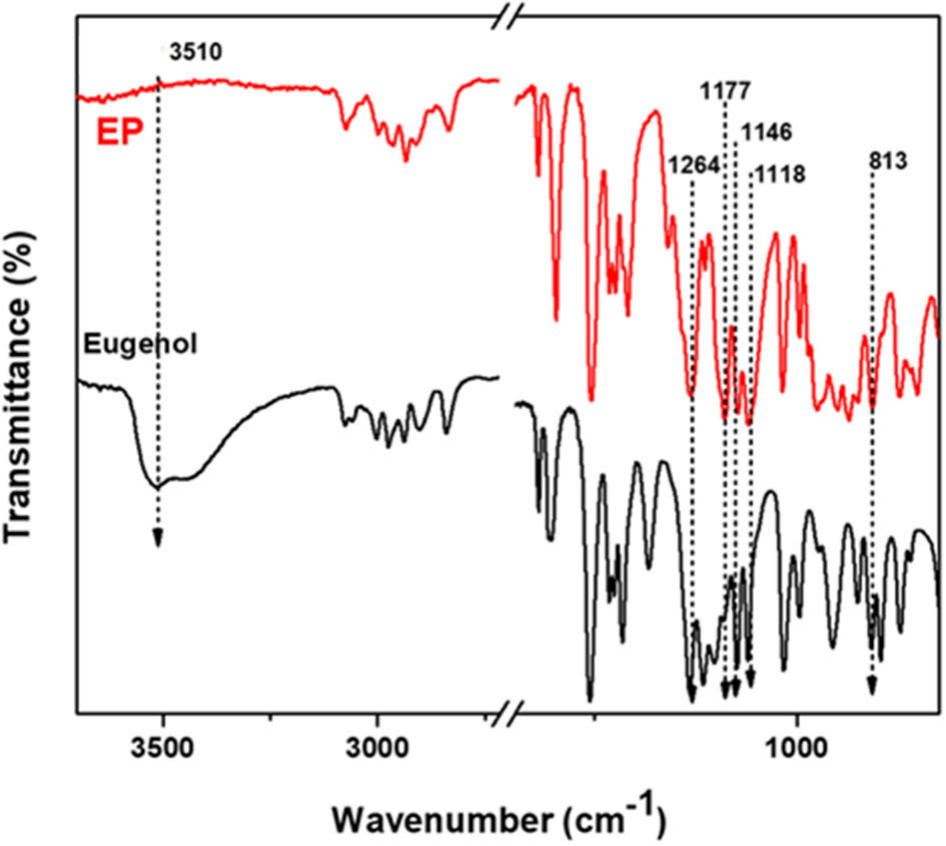
FTIR spectra of eugenol and EP.

Figure 5 and Supplementary Figure 4 show the FTIR spectra of EP, eugenol and CP, cardanol, respectively. The absorption bands due to the OH stretching of eugenol and cardanol, at 3,510 and 3,350 cm^−1^, were absent in the spectra of EP and CP, respectively. Concurrently, new peaks at 1,177, 1,146, 1,118 cm^−1^ and 1,204, 1,138 cm^−1^ in EP, and CP spectra, respectively, were assigned to P=N stretching of the phosphazene ring. The phenol attachment to the phosphazene was further evidenced by newly formed P-O-Ar, observed at 972 cm^−1^ in CP (P-O-Ar deformation vibration) and 950 and 813 cm^−1^ in EP (P-O stretching vibrations). Because of the difference in substitution on the benzene ring, there are slight differences in the peak wavenumbers of EP and CP (Allcock and Fuller, 1980; Krishnadevi et al., 2015; Shukla et al., 2015; Zhang et al., 2017; Amarnath et al., 2018a).

### Monitoring of Polymerization Reaction of XP (X = C or E) and C-trisapm

#### FTIR Spectroscopy

**Figure 6:**
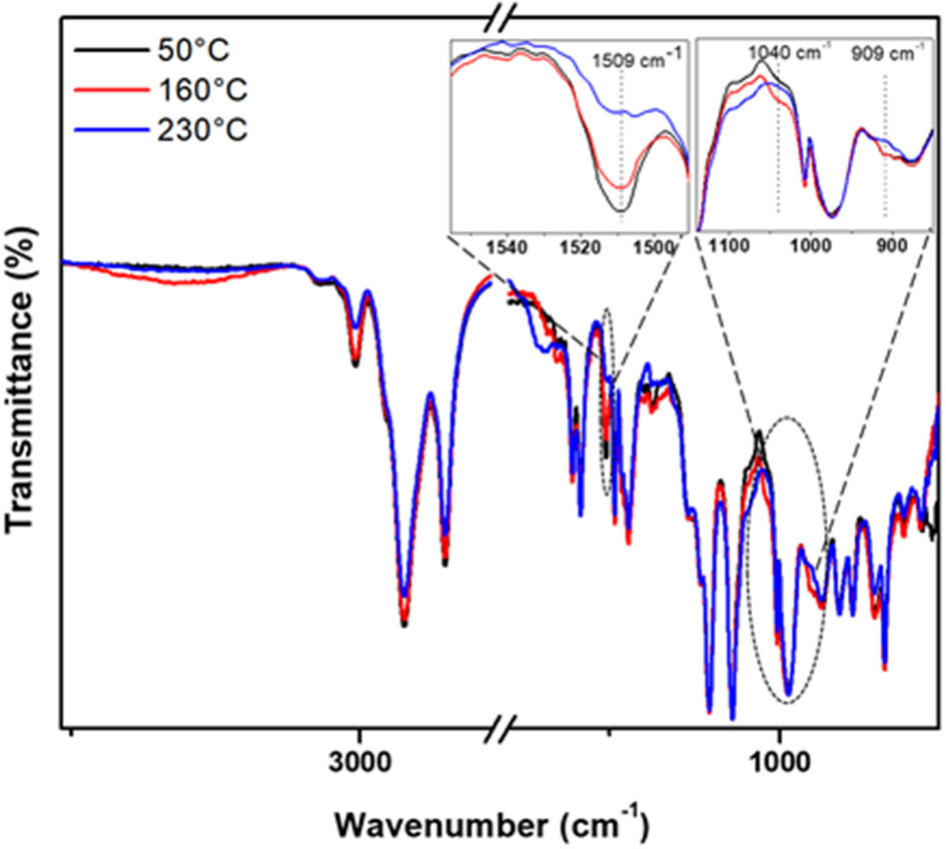
Normalized FTIR spectra for CP_1_T_3_ heated at 50 °C, 160 °C and 230 °C.

The non-isothermal heating of pristine CP/EP, C-trisapm, and their different blends (3:1, 1:1, 1:3), and changes were followed by FTIR spectroscopy. The spectrum of each sample was recorded after heating for 1 h at different temperatures. The characteristic peaks at ~1,240 and 1,041 cm^−1^ corresponding to the asymmetric and symmetric stretching of C-O-C due to benzoxazine ring in C-trisapm and undergoes ring-opening reaction, which is initiated when heated at ~200°C ≥ 2 h (Shukla et al., 2016). Due to significant overlap of former stretch with phosphazene core vibrations, it cannot be monitored in the blends with CP/EP. Furthermore, since the ROP of phosphazene ring occurs at ≥250°C, as reported by Allcock and Fuller (1980), which is far higher than our temperature of study, therefore P=N peak at 1,146 and 1,118 cm^−1^ can be used as an internal reference in FTIR study. Figure 6 and Supplementary Figure 6 show the normalized FTIR spectra to monitor and compare the conversion of one of the representative monomer blends based on CP and EP with C-trisapm, respectively. However, a significant decrease in intensity of the characteristic benzoxazine ring stretch at 1,040 cm^−1^ was found to decrease upon heating. To further support the oxazine ring-opening reaction, a simultaneous decrease in the intensity of peak at 1,510 cm^−1^, associated with a substituted benzene ring, was found to decrease confirming changes in the degree of substitution (see Figure 6 for FTIR spectrum of CP_1_T_3_) (Agag et al., 2010). On the contrary, EP/C-trisapm blends showed significant overlap in the characteristic oxazine ring-opening reaction with phosphazene core.

Additionally, as temperature increases, the intensity of the olefinic C-H and vinyl group stretching vibrations at 3,008 cm^−1^ and ~900 cm^−1^ decreases, in both the neat CP/EP and its blend with C-trisapm. This strongly supports a simultaneous involvement of the double in the crosslinking free radical reaction both inter- and intra-molecular within to form EP/CP/C-trisapm framework (Ma et al., 2017) and with oxazine ring (Amarnath et al., 2018b) *via* electrophilic reactions. A temperature dependent decrease with the nature of double bonds is observed, in neat CP and EP, which initiates above 100°C, and above 150°C, respectively. This suggests a higher reactivity of double bonds present in cardanol as compared to eugenol-based monomer under the aerobic conditions. This is due to the higher reactivity of double bonds in the former as they are allylic in nature, while double bonds in EP are isolated in nature.

**Scheme 2:**
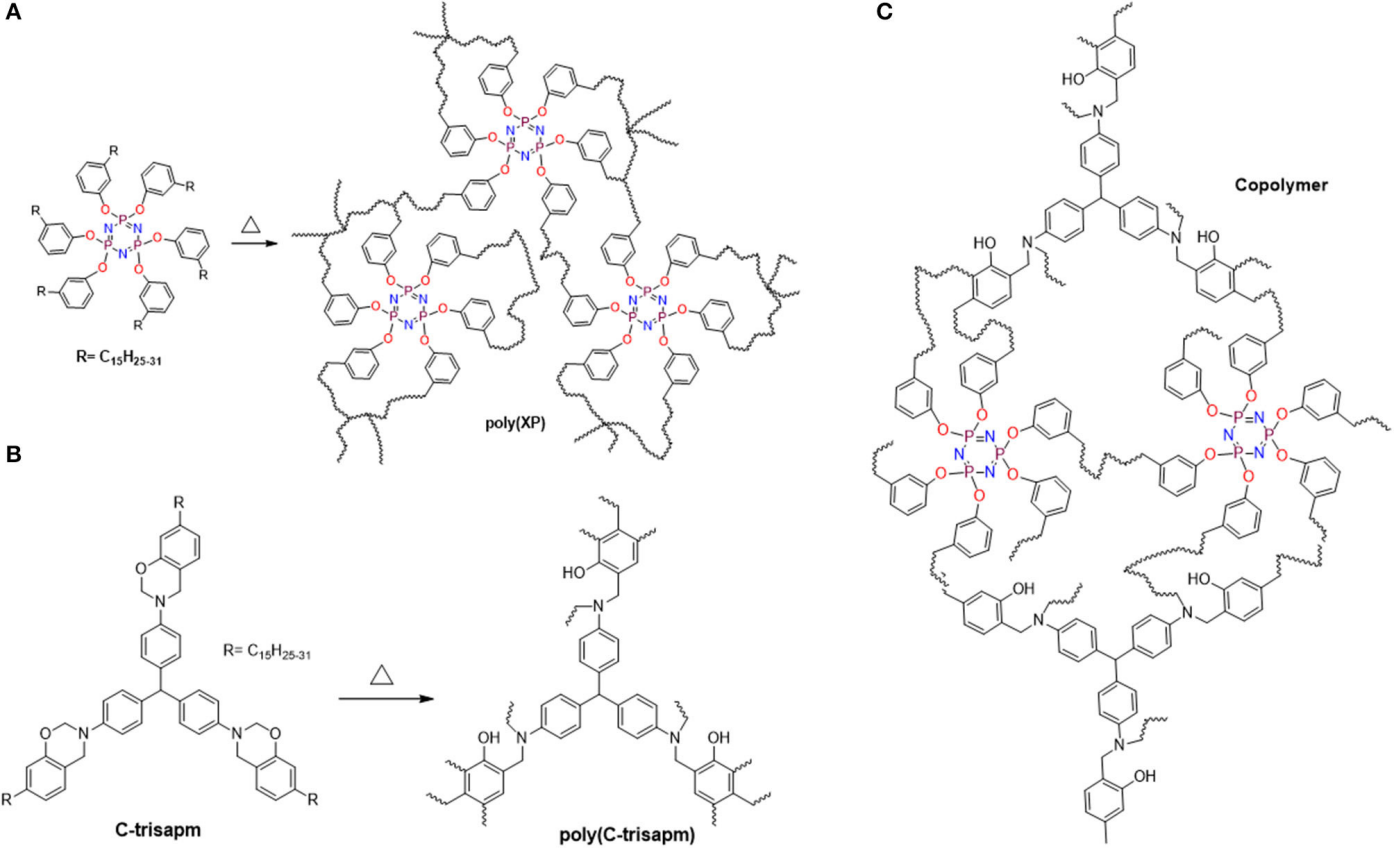
Probable polymerization reactions: **(A)** self-crosslinking via double bonds, **(B)** oxazine ring-opening polymerization, **(C)** Copolymerization: Co-reaction of double bonds and oxazine ring.

Additionally, the consumption of double bonds can also occur *via* co-reaction with an oxazine ring-opening reaction (Amarnath et al., 2018b). The above studies suggested the reactions possible are the usual ROP reaction of benzoxazine and co-reaction of alkylene double bonds inter- and intra-molecularly present in EP/CP/C-trisapm. The probable structure of a network is shown in Scheme 2.

#### Differential Scanning Calorimetry

**Figure 7:**
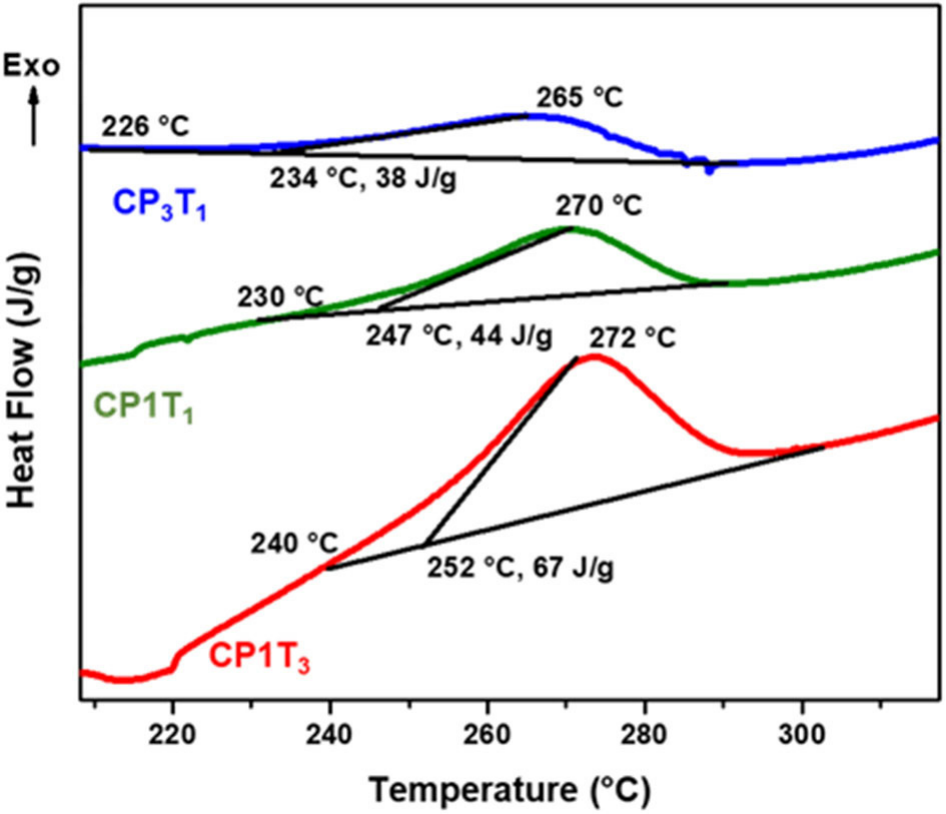
DSC thermograms of CP:C-trisapm monomer blends at different ratio, at a heating rate of 10 °C/min under N_2_ atmosphere.

The polymerization reaction of CP and EP blends with C-trisapm was followed by DSC, and the DSC scans are shown in Figure 7 and Supplementary Figure 7, and results are summarized in Table 1. DSC thermograms, in the case of CP/C-trisapm, revealed an increase in CP content in the blend causes a decrease in the initiation temperature (*T*_i_), the onset temperature (*T*_o_), the exothermic peak temperature (*T*_p_), and the heat of polymerization reaction (Δ*H*). Unlike in CP blends, an increase in the EP content in the C-trisapm blends results in higher values of *T*_i_, *T*_o_, and *T*_p_ values, whereas Δ*H* followed the same trends as it was in the case of CP blends. Interestingly, a slight decrease in polymerization temperature is observed in CP/C-trisapm under the studied conditions. These results are again supportive of higher reactivity of double bonds in CP than in EP, which is in congruence with FTIR results. A decrease in Δ*H* in both the blends could be attributed to the three reasons, first, lower reactivity of double bonds than oxazine ring, second dilution effect of oxazine functionality by the addition of XP monomer in the blend and third the analysis was performed under nitrogen conditions, which may prevent crosslinking reactions of double bonds. To confirm, the latter reason, DSC spectra of both neat CP and EP were recorded under the same conditions. No exotherm corresponding to polymerization of both EP and CP was noticed; EP showed an endotherm peak at 90°C, accounted to its melting transition as shown in Supplementary Figure 8. This confirms that the crosslinking reactions are mediated under aerobic conditions only.

**Table 1:** Thermal properties of CP, EP, C-trisapm, and their respective blends.

**Samples**	**T*_***i***_* (°C)**	**T*_***o***_* (°C)**	**T*_***p***_* (°C)**	**ΔH (J/g)**	**T_**10%**_ (°C)**	**T*_***max***_* (°C)**	**Char Yield at 800°C (%)**	***P* (%)**	**Renewable phenol (%)**	**LOI**	**Smoke density**
C-trisapm (T)	207	254	271	108	427	442, 482, 709	8.3	0	70.40	20.82	54.6
CP_1_T_3_	240	252	272	67	431.1	453, 482, 710	28.5	1.19	23.28	28.9	51.6
CP_3_T_1_	226	234	265	38	403.2	405, 506, 710	42.7	3.56	69.83	34.58	47.5
CP	–	–	–	–	352	386, 503	29.3	4.75	93.10	29.22	34.01
EP_1_T_3_	224	252	275	85	413.7	410, 487, 712	28.7	2.09	21.97	28.98	35.39
EP_3_T_1_	260	261	285	7	361	361, 481, 709	28.5	6.26	65.92	28.9	39.83
EP	–	–	–	–	301.6	355, 458, 712	32.4	8.34	87.89	30.46	18.80

#### NMR Spectroscopy

To probe the structural changes of the species formed during polymerization reactions, neat XP samples were heated under nitrogen atmosphere at 200°C for different time intervals and NMR was recorded of the soluble fraction. A noticeable decrease in solubility of the samples was observed with an increase in heating time. Hence, before any measurements were taken, the samples were left in the deuterated solvent for several hours. Supplementary Figures 9, 10 show the normalized stacked ^1^H NMR spectra of XP heated for different intervals.

We observed the appearance of a new set of signals of similar pattern and chemical shifts that matched significantly with those of corresponding phenol. The newly observed signals cannot be attributed to the formation of phenols as scission of C-O-P bond demands a high temperature under vacuum conditions, and are more susceptible to rearrangement reactions (Ferrar et al., 1980). Furthermore, phenols cannot be generated, otherwise, a significant decrease in the polymerization temperature of the oxazine ring would have been noticed (Ishida and Rodriguez, 1995).

The intensities of the new signals were found to increase with the time and with respect to the peak intensities of the unheated monomer. The new signals became prominent only after 3 h in CP vs. 6 h in EP, with increasing signal intensity of new signals and a concomitant decrease of signals in parent monomer with heating time. Some important changes were observed in the aromatic region 6.6–7.2 ppm, as well as in the 5–6 ppm region, the proton of the unsaturated chains of cardanol. The peaks at 5.87–5.77 and 5.07–4.97 ppm showed a decrease in intensity compared to the reference peak at 2.46 ppm and 48 h of heating. These peaks are no longer visible, indicating that these protons are no longer in the same environment. This change might therefore be attributed to the crosslinking reactions of the double bonds that may have resulted in the observed change in chemical shift values. Moreover, the reduced solubility of the heated samples further confirms the generation of a highly cross-linked mass. Furthermore, analysis to determine the structure of species, are under consideration.

Manjula et al. (1992) and John et al. (2019) reported the nature of chain reaction polymerization of cardanol. However, the antioxidant properties of cardanol showed its inability to undergo polymerization by free-radical initiators. However, oligomerization of cardanol without any catalyst (Rodrigues et al., 2006) or with acidic catalyst (Manjula et al., 1992) was observed. In our case, phenolic-OH was not present, as it was consumed in co-reaction with P_3_N_3_Cl_6_ and therefore an enhanced tendency in its involvement in polymerization reactions. Although a double bond in the propenyl group in EP may be less reactive through this mode of polymerization, their consumption occurs *via* co-reaction with oxazine ring-opening reaction (Amarnath et al., 2018b).

In the aliphatic region, new signals were also observed with the disappearing of the existing ones. For instance, a triplet was formed at 2.55 ppm that might be due to the shifting of the triplet at 2.44 ppm, corresponding to the benzylic proton. This shift may be attributed to the change in the environment, with the protons of the new species formed sharing a similar environment to the crude cardanol.

For a more thorough study, the curing kinetic experiment was repeated in the presence of air. The development of the new species was also found to take place under aerobic conditions, but the rate of formation of the new peaks was slower than under inert atmosphere. After 24 h of heating, the ratios of CP to the new species were 1:1 for the sample heated under nitrogen, and 1:0.75 for the sample heated in air, stating that the dissociation of CP into the new species was around 25% slower in air.

We may here conclude from the heating kinetic study of CP that two possible reactions might be happening that will account for the observations made. Firstly, the double bonds are being consumed through crosslinking reaction and secondly, the opening of the phosphazene ring may have happened, and as reported earlier, tautomerism takes place, where the phenyl ring connected to the oxygen was bonded to the nitrogen, hence causing changes in the chemical shift values (Amarnath et al., 2018a).

However, after 48 h of heating, the heated sample became insoluble and hence the NMR was not recorded. For the heating time from 6 to 36 h, similar observations were made as CP, with the formation of new peaks were accompanied by a decrease in intensity of the starting EP peaks, and the chemical shifts of the developing peaks were very similar to those of eugenol. For instance, next to the peak at 3.27 ppm, which was used as a reference, a new doublet at 3.32 ppm was observed, which appears at almost the same chemical shift as the eugenol peak at 3.33 ppm. These changes in chemical shift values can be attributed to the shifting of the phenyl ring onto the nitrogen arising from the opening of the phosphazene ring. Unlike the case of CP, the double bond was not consumed, stating that crosslinking did not take place, but a slight downfield shift of the multiplet was observed, closer to that of eugenol. Therefore, like CP, it can be concluded that a new molecule was formed upon the heating of EP that was very similar to the starting eugenol.

**Figure 8:**
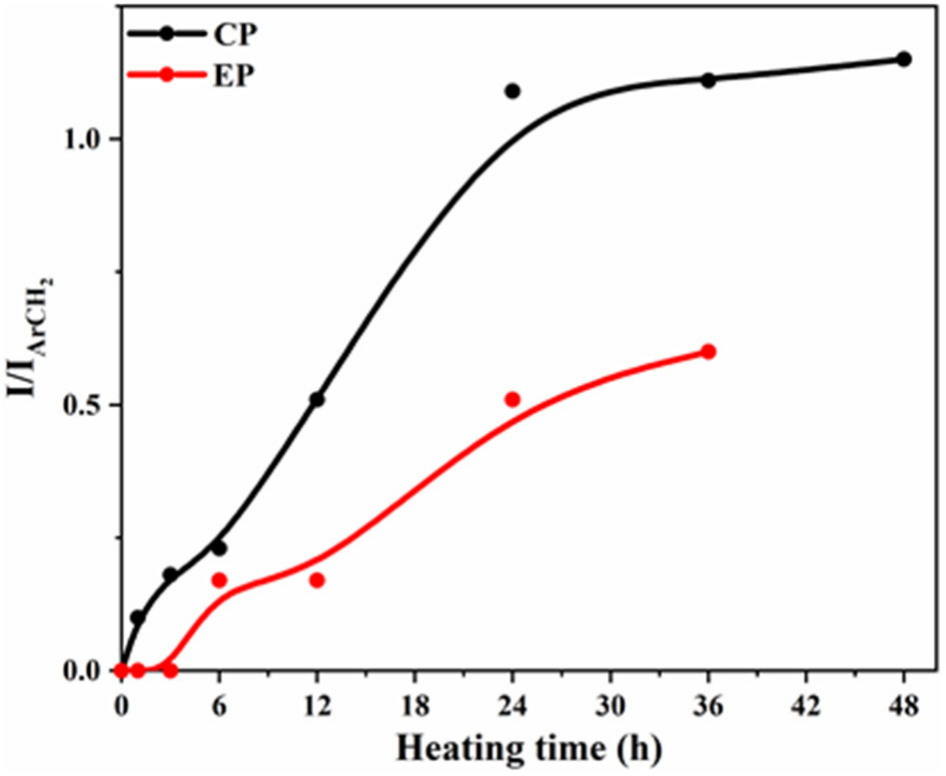
Variation of intensity ratios of ^1^H NMR signals against heating time for CP and EP monomer.

For both XP, the integration ratios of the existing and new peaks were plotted against heating time, as shown in Figure 8. For CP, the ratios of integration of the new peak at 2.55 ppm and that of the existing one at 2.46 ppm were plotted with the heating time and for EP, the ratios of the intensity of the new peak at 3.32 ppm to that of the original peak at 3.27 ppm were plotted against heating time. The formation of the new species occurs about twice as fast for CP as for EP.

### Thermal and Flame Retardant Analysis of the Polymers

**Figure 9:**
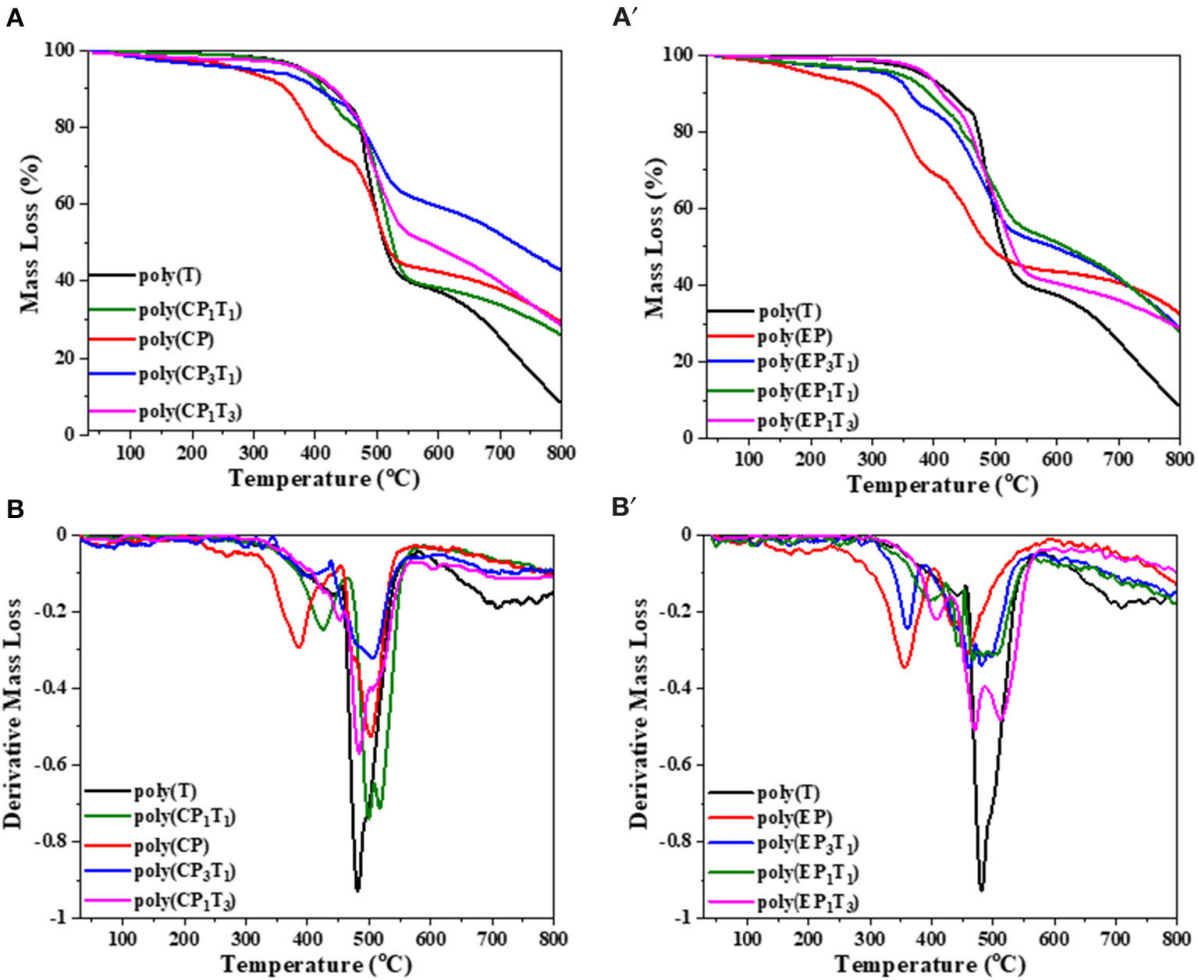
TGA **(A,A′)** and DTG **(B,B′)** traces of pristine polymers and their blends recorded at a heating rate of 20 °C/min.

Enhanced thermal stability of the polymer network of blends was observed, as was the pristine monomer from the TGA and differential thermogravimetry (DTG) traces, shown in Figures 9A,A′,B,B′ respectively. Both *T*_10%_ and *T*_max_ of the different polymerized blends were found to decrease in this order, CP_1_T_3_ > EP_1_T_3_ > CP_3_T_1_ > EP_3_T_1_. The thermal stability was found to be optimum at the 1:3 (XP: C-trisapm) ratio. This is due to a higher molar excess of C-trisapm in relation to XP monomers (1:2.6 for EP:C-trisapm and 1:4.6 for CP:C-trisapm) among the studied compositions, suggesting a higher crosslinking density of the resultant copolymer framework.

The thermal stability of copolymers was found to be optimum at a 1:3 (XP: C-trisapm) weight ratio. This is due to a higher molar excess of C-trisapm in relation to XP monomers (1:2.6 for EP:C-trisapm and 1:4.6 for CP:C-trisapm) among the studied compositions, suggesting a higher crosslinking density of the resultant copolymer framework.

A further increase in XP content in the blend decreased the initial thermal stability. However, both char yield, LOI value, and smoke density were found to decrease with an increase in phosphorous percentage, inferring the role of the phosphazene framework in properties such as flame resistance and smoke reduction. The char yield of the neat poly(C-trisapm), poly(CP), and poly(EP) are 8%, 29%, and 32%, respectively. The incorporation of XP in C-trisapm resulted in a significant improvement in %char yield of the polymer blends as compared to pristine poly(C-trisapm). The blends poly(CP_1_T_3_), poly(EP_1_T_3_), and poly(EP_3_T_1_) show char yield of 28%, while poly(CP_3_T_1_) shows an exceptionally higher char yield of 42% among all the synthesized polymers. The higher char yield of the blends arises as a result of the formation of intumescent char, characteristic of P-N, making phosphazene-rich as a good charring agent (Amarnath et al., 2018a). It must be noted that poly(CP_3_T_1_) has a much higher char yield than both pristine poly(C-trisapm) and poly(CP), suggesting that a significant role is played by crosslinking reactions between the two monomers. As expected, EP based blends showed lower thermal stability than CP, suggesting cardanol has a more pronounced participation in crosslinking reactions. At around 400°C, besides decomposition of polybenzoxazine framework, even the polyphosphazene network may decompose to release amines, phosphate, and related compounds according to the literature (Maynard et al., 1991). Thermally-induced intramolecular migration of the phenyl group of cardanol and eugenol may take place in phosphazene due to P-O-C cleavage (Ferrar et al., 1980; Hayes and Allen, 2016). This decomposition results in the mass loss as observed in the DTG, with the highest mass losses occurring for pristine phosphazene frameworks of poly(XP) as they bear the highest percentages of the P-O-R linkages. However, these mass losses are significantly minimized showing synergism between the polybenzoxazine and polyphosphazene frameworks in the blends. The percentage of renewable phenol content was extremely high in all of the synthesized polymers, supporting the green potentials of the used monomers.

Limiting oxygen index (LOI) measures the minimum oxygen concentration required to support combustion is calculated from the char yield. A compound with an LOI value >20.9%, the percentage of oxygen in the air, is considered as a flame retardant since it requires higher oxygen concentration to burn. An LOI in the range of 21–28 indicates slow burning rates, with a range of 28–100 for self-extinguishing materials. The LOI of the different polymers are presented in Table 1. The LOI value of poly(C-trisapm) changed from 20 to higher values in the blends. Incorporation of 1.1% of P in C-trisapm showed slow burning characteristics and all the other polymers showed self-extinguishing characteristics. The data showed that the higher the percentage of phosphorus in the polymer, the higher the LOI and hence better retardancy, indicating that the addition of phosphorus to C-trisapm results in the improvement of flame retardancy (Lu and Hamerton, 2002)^1^.

**Figure 10:**
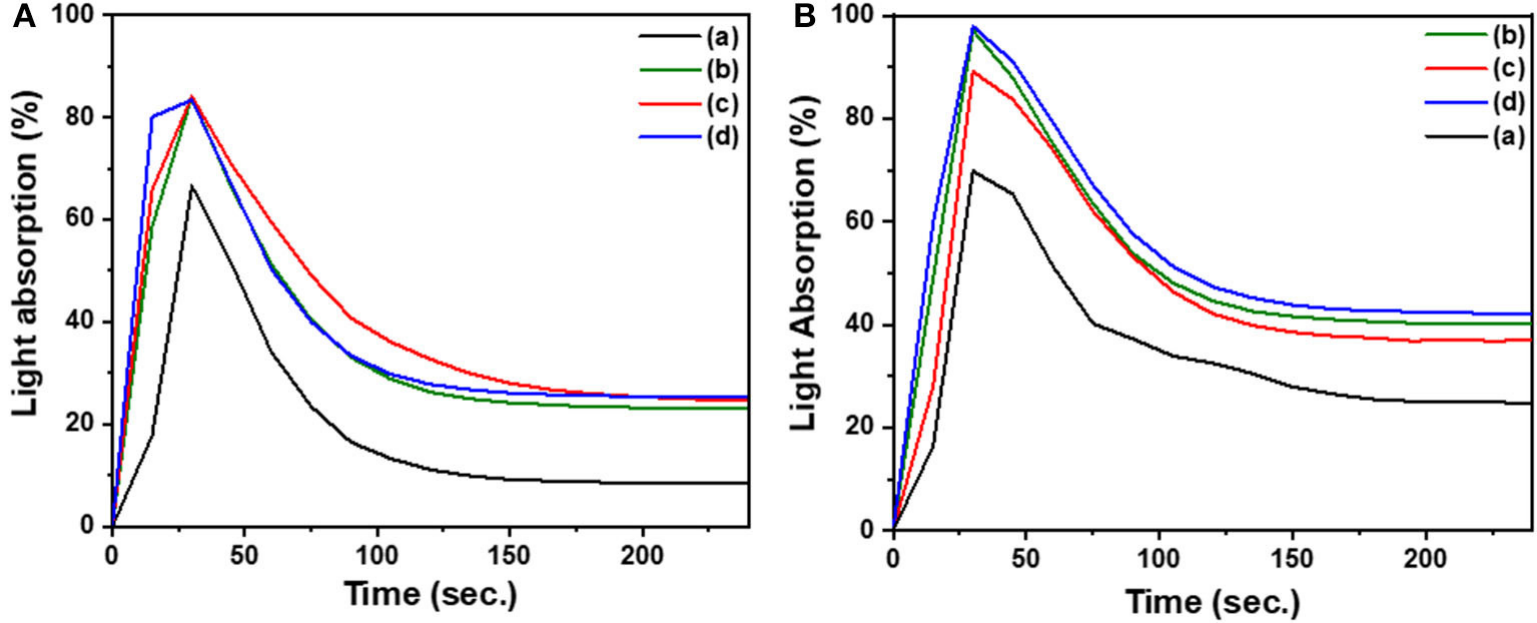
Plots of light absorption by sensor with time during burning of samples (a) XP, (b) X_1_T_3_, (c) X_3_T_1_, (d) C-trisapm. Plot **(A)** is for CP and **(B)** is for EP.

Smoke density is a means of measuring the relative optical amount of smoke released by a burning sample. Measurements are made by burning the sample in a smoke density chamber, consisting of a light source and a photometer situated on opposite sides of the chamber. The attenuation of the light beam is determined by the accumulation of smoke from the burning sample in the enclosed chamber. From this experiment, the light absorption can be plotted vs. time and the rate of smoke production is determined from the area under the curve (Lyons, 1975). To understand the effect of the incorporation of a phosphorous rich reactive additive to poly(C-trisapm), the relative smoke emission of the different sample smoke emission was evaluated. The measurements from the optical system are represented in the plots of light absorption against time in Figures 10A,B. The area under the curves was calculated and reported in Table 1. The smoke density data, like the LOI data, was found to be dependent on the phosphorus content in the sample. C-trisapm, which contains no phosphorus, burns with a smoke emission of 37.80%, whereas CP and EP with phosphorus contents of 4.75 and 8.34% produced 34.01 and 18.80% smoke density, respectively. This revealed a substantial reduction in smoke due to phosphazene moiety incorporation in the polymers.

UL (Underwriters Laboratories) tests are burning tests that evaluate the flammability of polymers. The UL-94 test is a vertical burn test that determines the vertical burning characteristics of a polymer. The flammability is rated from V0 to V2, with V0 being the best flame retardancy achieved when burning stops within 10 s after application of two ignitions of 10 s each to the sample, with no dripping (Lu and Hamerton, 2002). Poly(C-trisapm) sample burnt instantaneously with a much shorter combustion time and noticeable drippings, which is in congruence with LOI results. The flame resistance of the blends is evident from the digital images of phosphazene containing polymers before and after burning, shown in Supplementary Figure 11. Pure poly(CP) and its blends did not catch fire instantaneously, unlike poly(C-trisapm). The flame resistant characteristics of crosslinked CP/C-trisapm blends was found to increase with an increase in phosphorus content, which corroborates with LOI results. Neat EP homopolymer and its copolymer with C-trisapm cannot be fabricated into vertical specimens as they were found to be very brittle, due to rigidity of the framework and the C-3 propylene chain of EP. The internal plasticization effect of the C-15 long alkylene chain in CP may be responsible for the induced flexibility of the fabricated polymer that allowed the above analysis in the case of CP/C-trisapm copolymer.

**Figure 11:**
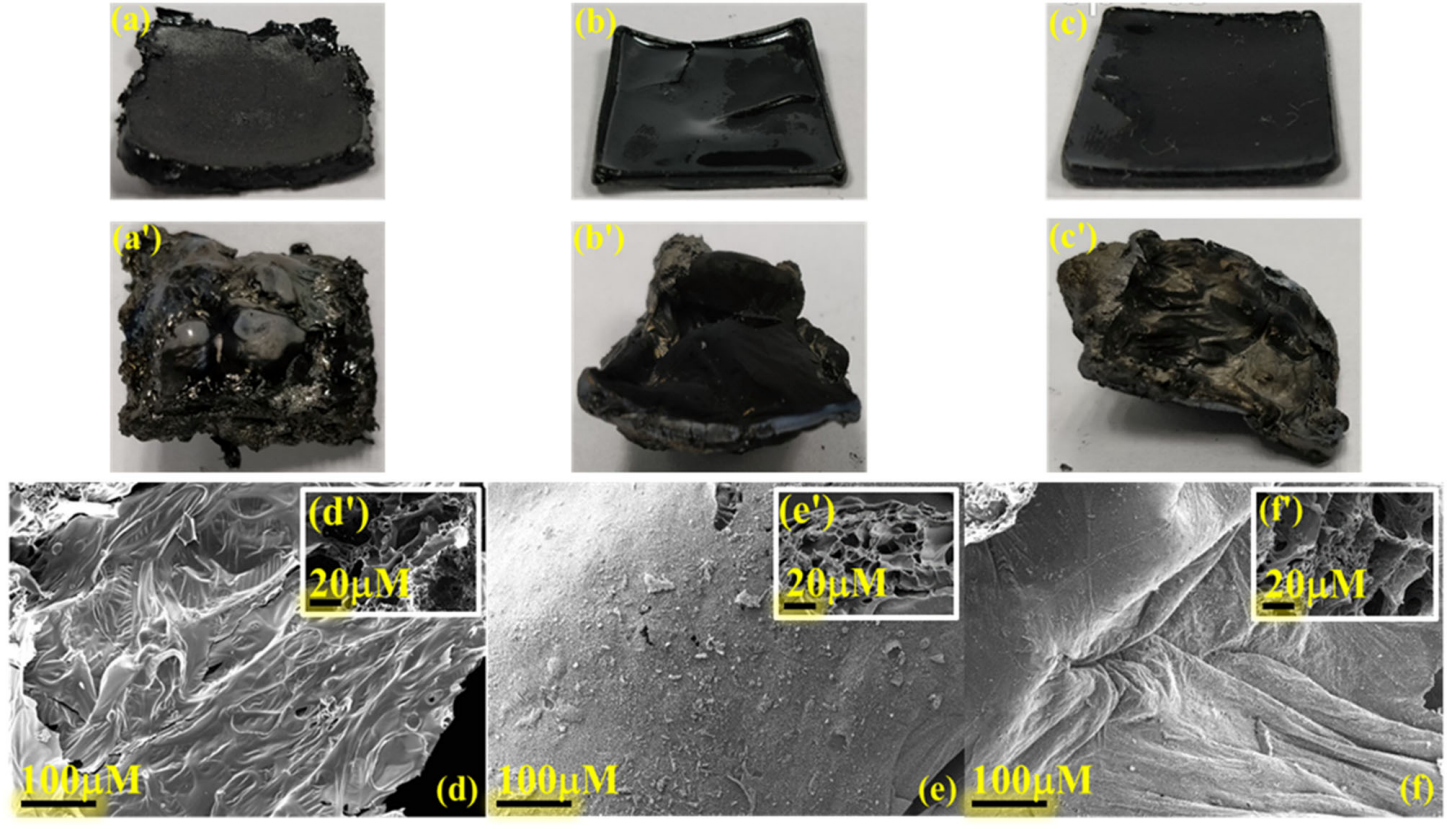
Digital images of cured samples [l × w × h: (25.0 ± 0.1) x (25.5 ± 0.1) × 3.0 mm] of (a) poly(CP), (b) poly(CP_3_T_1_), (c) poly(CP_1_T_3_) before (a, b, c) and after burning (a′, b′, c′); SEM images of poly(CP), (b) poly(CP3T1), (c) poly(CP_1_T_3_) surfaces of residual char: exterior (d, e, f) and interior (as inset) (d′, e′, f′), respectively.

Digital images of thermally cured samples before and after smoke density analysis are shown in Figures 11a-c′. The CP homopolymer showed a rough contour surface, suggesting rigidity of polymer framework. However, its copolymer with C-trisapm showed appreciable molding characteristics, suggesting benefit imparted by benzoxazine monomer to phosphazene monomer. The burnt sample digital images revealed an intensive expansion of the polymer matrix and a significant formation of protective charred layered architecture. These results are consistent with the intumescent flame retarding mechanism of phosphazene structure.

Figures 11d–f′ shows the SEM images, which determine the morphology of both the exterior and interior surface of the char residue obtained after smoke density measurements. With an increase in CP content, the exterior surface morphology changed from a rippled to a smooth surface, while interior surface morphology showed an increase in the development of a network of porous honeycombed microstructures separated by thin layers of boundary. The appearance of compact char residue with a profound interconnected network of lacunae accounts for flame mitigation strategy, which is associated with the phosphazene rich polymer network. On the contrary, the formation of larger cracks on the exterior surface of the order of 20–30 μm observed in char of poly(EP_3_T_1_), Supplementary Figure 12b. The cracks reduced substantially with the increase in C-trisapm content, as noticed from the exterior surfaces of poly(EP_1_T_3_). The analysis of interior morphology also showed the formation of big porous microstructures dependent on EP content. The formation of bubbles and cracks is accounted to the rigid outer coating in the case of EP containing copolymers, which may have burst to release the exchange of heat and air (oxygen), thereby inhibiting the advancement of flame as a structural safety measure. Energy dispersive spectroscopy (EDS) analysis, Supplementary Table 1 clearly shows a relatively higher atomic % ratio of experimental P/O to that of theoretical results. This further supports the development of P and O rich domains to form polyphosphoric acids, which enable carbonization reaction to form heat resistant char, by acting as a dehydrating agent at high temperatures (Ma and Fang, 2012).

To explore the lowering in thermal stability upon XP incorporation in C-trisapm monomer, we performed swelling studies of neat polymers/polyphosphazene polybenzoxazine copolymers. The polymer samples were kept in four different solvents (H_2_O, DMSO, EtOH, and CHCl_3_) and mass variation of samples was observed after every 24 h for four consecutive days. The relative swelling ratios are shown in Supplementary Figure 13. After soaking the polymers in solvents, the polymer was wiped with clean tissue paper to remove excess solvent from the surface. The samples were weighed immediately in a weighing balance with an accuracy of 10^−4^ g. The polymer samples in chloroform were found to disintegrate (with no solubilization) in all the cases after 24 h, hence further studies were not carried out using this solvent. It was observed that the swelling ratio of neat polymer and copolymers remains unaffected in water and ethanol. We observed a lower swelling ratio in water than in ethanol followed by DMSO, suggesting the very high hydrophobicity of the samples. In DMSO, incorporation of XP in C-trisapm increased the swelling ratio, suggesting a lowering in crosslink density. The value is found to be greater in the case of EP than CP based polymers, which corroborates the TGA studies.

## Conclusions

This study has reported on the synthesis and characterization of abundantly available agro-origin source phenols, cardanol, and eugenol, based reactive flame retardant containing a phosphazene core. The hexafunctional nature of alkylene units with phenyl spacer to phosphazene core ensures appreciable reactivity of double bonds in inter- and intramolecular fashions with the CP/EP monomers and C-trisapm blends. The co-reaction between the monomers was supported by a simultaneous reduction of double bonds and an oxazine ring by FTIR spectroscopy. Newly formed species as a result of aerobic oxidation were noticed by NMR spectroscopy. Additionally, the CP alkylene chain was found to be more reactive than the propylene unit in EP as the former is allylic vs. non-conjugated. Swelling ratio studies have suggested a very high hydrophobicity of the polymers allowing their capability to withstand aqueous conditions for a prolonged duration. However, incorporation of XP in C-trisapm showed a marginal increase in swelling ratio, confirming a lowering in crosslink density than neat poly(C-trisapm). This change in value is slightly higher in the case of EP than CP based copolymers, supporting higher reactivity of the latter in crosslinking reactions. The incorporation of both the monomers has substantially improved flame resistant characteristics as supported by TGA, LOI, UL-94, smoke density, and SEM analysis. The presence of oxazine functionality, double bonds, high renewable content, and a phosphazene core is encouraging for their utility not only to benzoxazine chemistry but also in many other polymers. The easy one-step synthetic method of both the EP and CP monomers holds great potential as a replacement for many other environmentally toxic flame-retardants. This study is the first report using the phosphazene core containing cardanol and eugenol units as reactive type flame-retardants to exhibit high flame retardation efficiency and reactivity with the oxazine core. These findings may be useful in designing future generations of safer and eco-friendly reactive flame-retardants based on abundantly available synthons.

## Data Availability Statement

All datasets generated for this study are included in the article/Supplementary Material.

## Author Contributions

BL conceived the concept and designed the structures. DA and NA performed the synthesis and characterization of data. All authors thoroughly discussed the analysis of the data and contributed to the writing of the manuscript.

## Conflict of Interest

The authors declare that the research was conducted in the absence of any commercial or financial relationships that could be construed as a potential conflict of interest.

## Footnotes

^1^Available online at: http://www.uow.edu.au (accessed October, 2009).
