# Supplementary material for: Cardanol and Eugenol Sourced Sustainable Non-halogen Flame Retardants for Enhanced Stability of Renewable Polybenzoxazines
Source: Front Chem. 2020 Sep 30;8:711. doi: 10.3389/fchem.2020.00711 (PMC7554585; doi:10.3389/fchem.2020.00711)
Supplement: Supplementary file 1 [file Presentation_1.pdf]

Supplementary Material

Cardanol and Eugenol Sourced Sustainable Non-Halogen Flame Retardants for Enhanced Stability of Renewable Polybenzoxazines

Divambal Appavoo, Nagarjuna Amarnath, Bimlesh Lochab*

Materials Chemistry Laboratory, Department of Chemistry, School of Natural Sciences, Shiv Nadar University, Gautam Buddha Nagar, Uttar Pradesh 201314, India

***Correspondence:**
Bimlesh Lochab
bimlesh.lochab@snu.edu.in

Total 15, Figure 12, Table 1

Synthesis of C-trisapm-----S2

Scheme S1. Synthesis of C-trisapm benzoxazine monomer.-----S3

Figure S1. ^{1}H NMR spectra of crude cardanol and CP (Solvent*: $CDCl_3$)-----S3

Figure S2. ^{13}C NMR spectra of crude cardanol and HCPP (Solvent*: $CDCl_3$)-----S4

Figure S3. ^{31}P NMR spectra of $N_3P_3Cl_6$ and CP (Solvent*: $CDCl_3$)-----S4

Figure S4. FTIR spectra of crude cardanol and CP. -----S5

Figure S5. ^{1}H NMR spectrum of C-trisapm-----S6

Figure S6. Normalized FTIR spectra for EP_1T_3 heated at 50 °C, 150 °C and 200 °C. -----S7

Figure S7. DSC thermograms of EP:C-trisapm monomer blends at different ratio, at a heating rate of 10 °C/min under N_2 atmosphere. -----S8

Figure S8. DSC thermograms of EP and CP monomer at a heating rate of 10 °C/min under N_2 atmosphere. -----S9

Figure S9. Stacked ^{1}H NMR spectra of cardanol and CP. The monomer CP was heated at 200 °C, under N_2, for different time intervals. Peaks normalized at 2.46 ppm. -----S10

Figure S10. Stacked of ^{1}H NMR spectra of eugenol and EP. EP monomer was heated at 200 °C, under N_2, for different time intervals. Peaks normalized at 3.27 ppm.-----S11

Figure S11. Digital images of the samples which passed the vertical burning test (before and after analysis) (a, a') poly(CP), (b,b') poly(CP_3T_1), (c, c') poly(CP_1T_3), and (d, d') poly(C-trisapm).-----S12

Figure S12. SEM images of external and interior (inset) surfaces of residual char of (a) poly(EP), (b) poly(EP_3T_1), (c) poly(EP_1T_3).-----S13

Table S1. Relative atomic % for P and O element before and after burning, determined from EDS analysis.-----S13

1 Synthesis of C-trisapm

1.1 Synthesis of *tris*-apm

tris-p-Aminophenylmethane (tris-apm). To a solution of *p*-rosaniline hydrochloride (0.50 g, 1.54 mmol) prepared in ethanol (40 mL), sodium borohydride (0.53 g, 14.00 mmol) was added in two lots with continuous stirring in an ice bath. Reaction continued till complete dissolution of sodium borohydride accompanied by a colour change from deep pink to yellow. Reaction was quenched with addition of water (75 mL) and a yellow precipitate of tris-apm was obtained after simple filtration. Yield 84 %. FTIR-ATR (diamond crystal/cm^{-1}): 3336, 3203, 3029, 1608, 1507, 1267, 815, 770; ^{1}H-NMR (500 MHz, $CDCl_3$, δ ppm): 6.88 (d, 6H, ArH), 6.60 (d, 6H, ArH), 5.24 (s, Ar_3C-H), 3.56 (br, s, 6H,–NH_2); LC-MS (ESI Interface-positive ions): $[M+H]^+$ 290.1651 (290.1613).

1.2 Synthesis of C-trisapm

Cardanol (1.56 g, 5.18 mmol), paraformaldehyde (0.31 g, 10.36 mmol), tris-apm (0.50 g, 1.72 mmol) was heated at a temperature of 90 ^oC for 5 h. On cooling, water (40 mL) was added and organic layer was extracted with chloroform (30 mL). The organic layers were combined and washed with NaOH (0.5 N, 100 mL) followed by washing with water (3 x 30 mL), dried over sodium sulphate and filtered. The solvent was removed under reduced pressure to give C-trisapm as viscous brown oil. Yield 80%; FTIR-ATR (diamond crystal/cm^{-1}): 3007, 2923, 2852, 1657, 1610, 1508, 1256, 1240, 1198, 1041, 1014, 990, and 960; ^{1}H-NMR (500 MHz, $CDCl_3$, δ ppm): 4.46 (s, Ar-CH_2-N-), 5.32-5.38 (m, CH=, CH_2=CH-, -OCH_2N-, HC=CH_2, Ar_3C-H); ^{13}C-NMR (125 MHz, $CDCl_3$, ppm): 50.32 ($ArCH_2N$–), 54.5 (Ar_3CH), 79.52 (–OCH_2N–); LC-MS (ESI Interface-positive ions) $[C\text{-trisapm}]^+$: 1261.8551, 1262.8649, 1263.8689, 1264.8783, 1265.8816, 1266.8904, 1267.8904, 1268.8966 (monoene: 1267.9408, diene: 1261.8938, triene: 1255.8469).

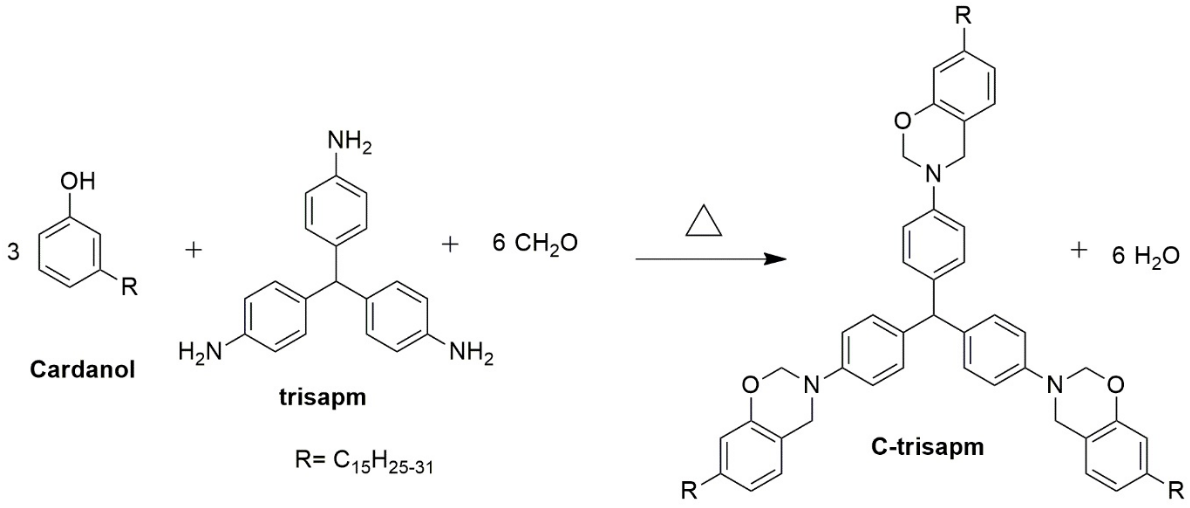

Scheme S1. Synthesis of C-trisapm benzoxazine monomer.

1.3 Supplementary Figures and Table

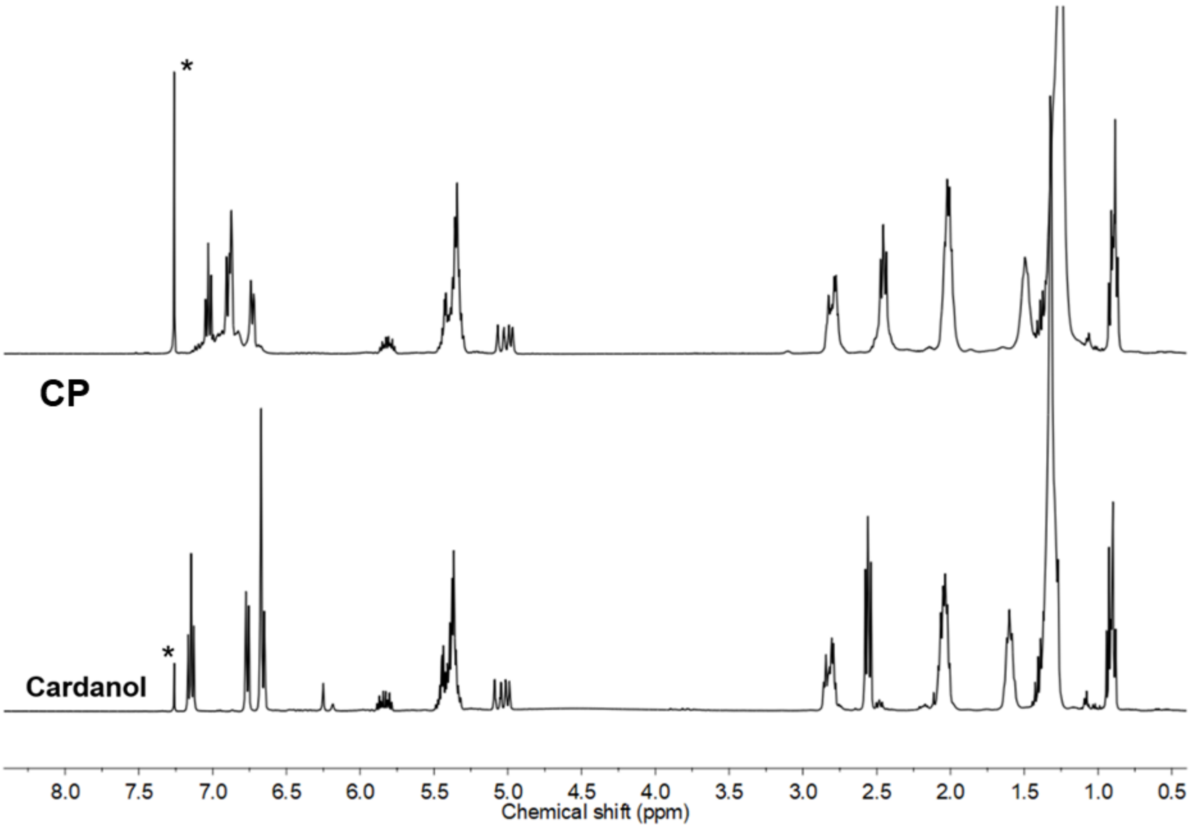

Figure S1. Stacked ^1H NMR spectra of crude cardanol and CP (Solvent*: $CDCl_3$)

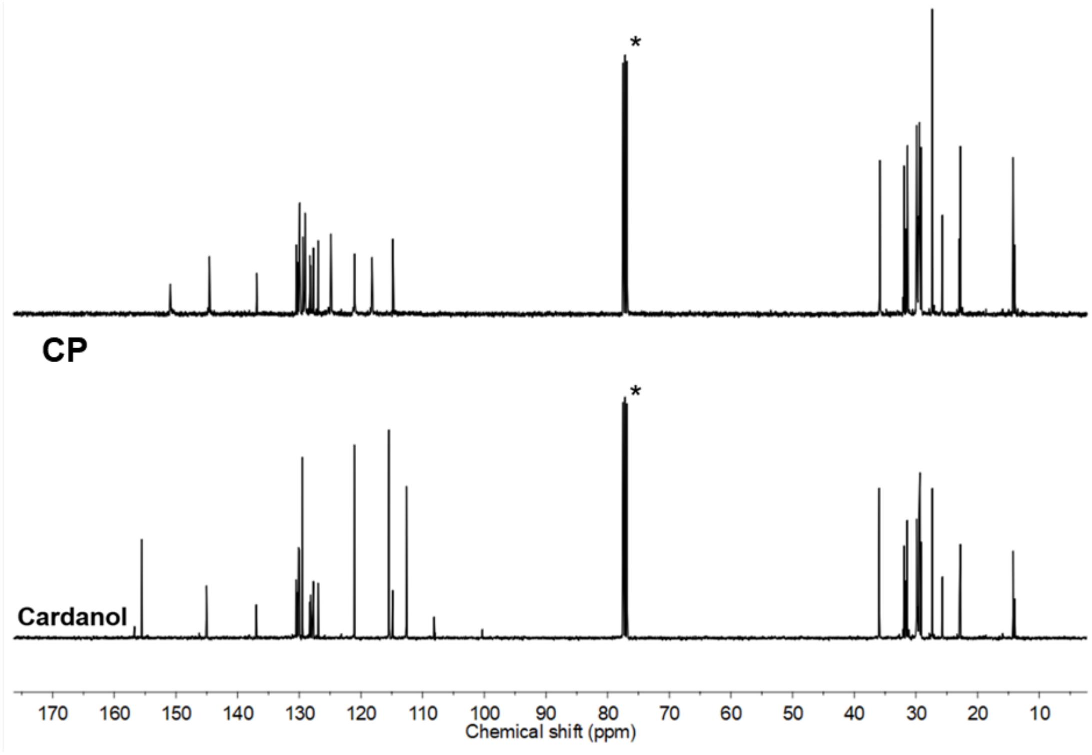

Figure S2. ^{13}C NMR spectra of crude cardanol and CP (Solvent*: $CDCl_3$)

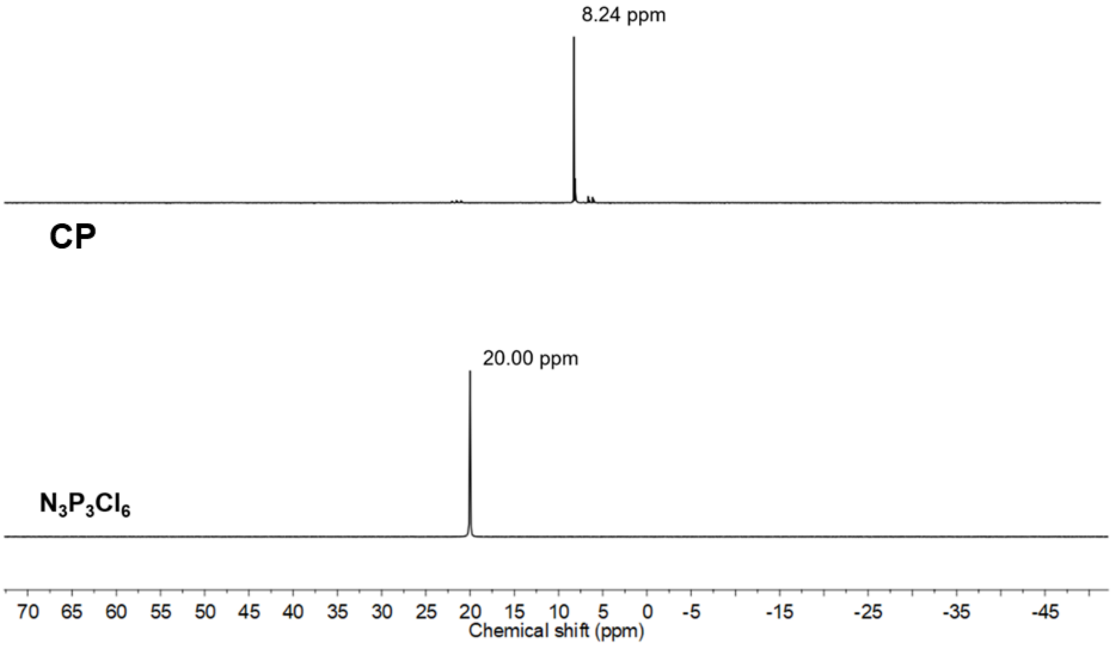

Figure S3. ^{31}P NMR spectra of $N_3P_3Cl_6$ and CP (Solvent*: $CDCl_3$)

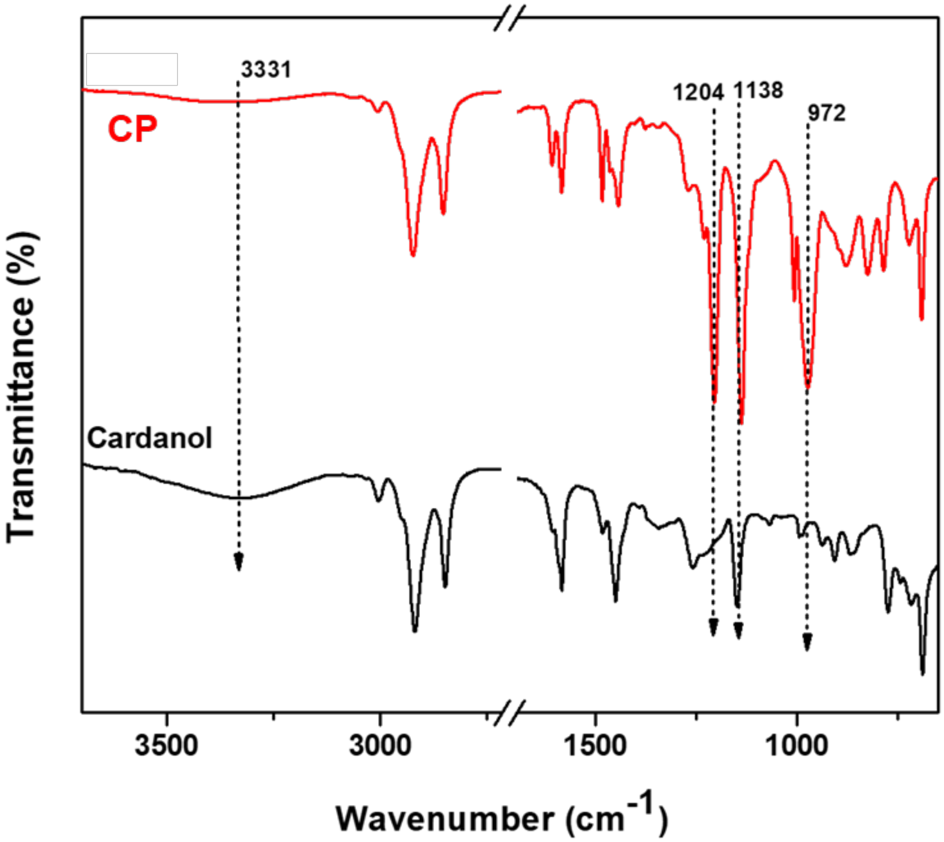

Figure S4. FTIR spectra of crude cardanol and CP.

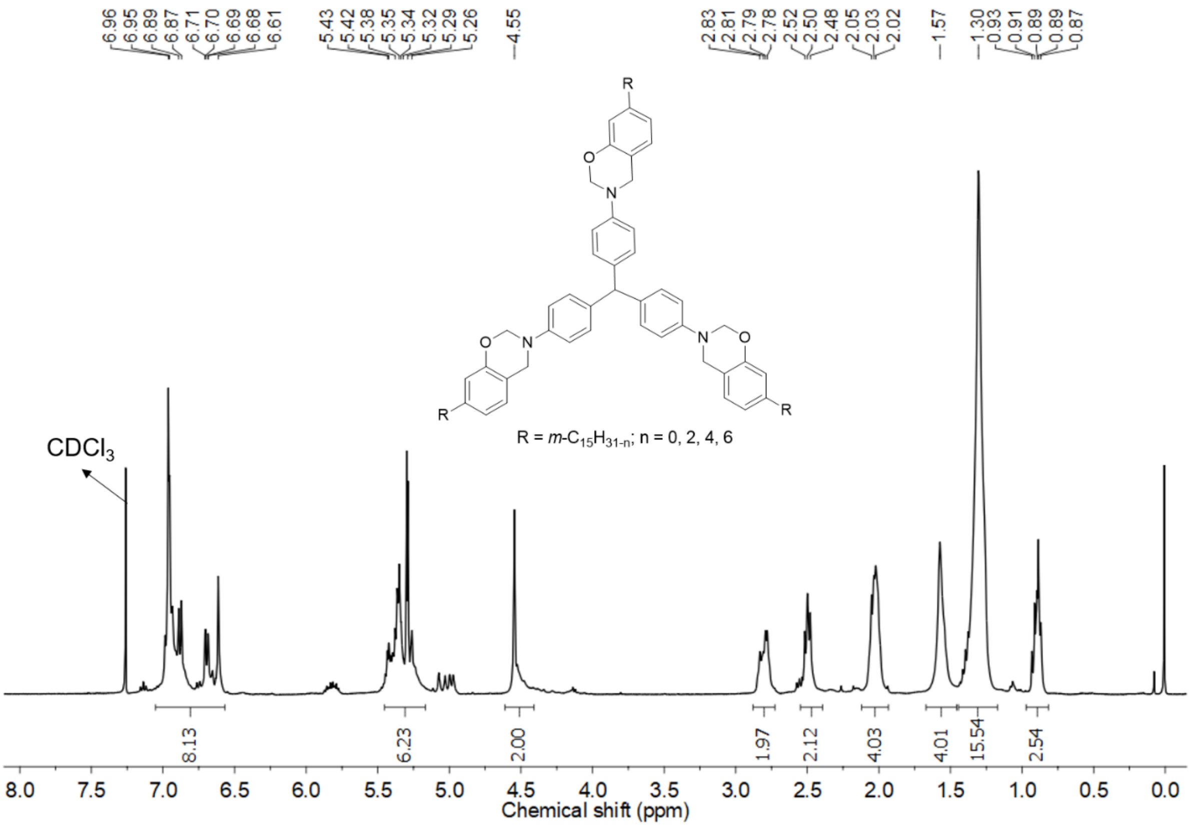

Figure S5. ^{1}H NMR spectrum of C-trisapm in $CDCl_3$.

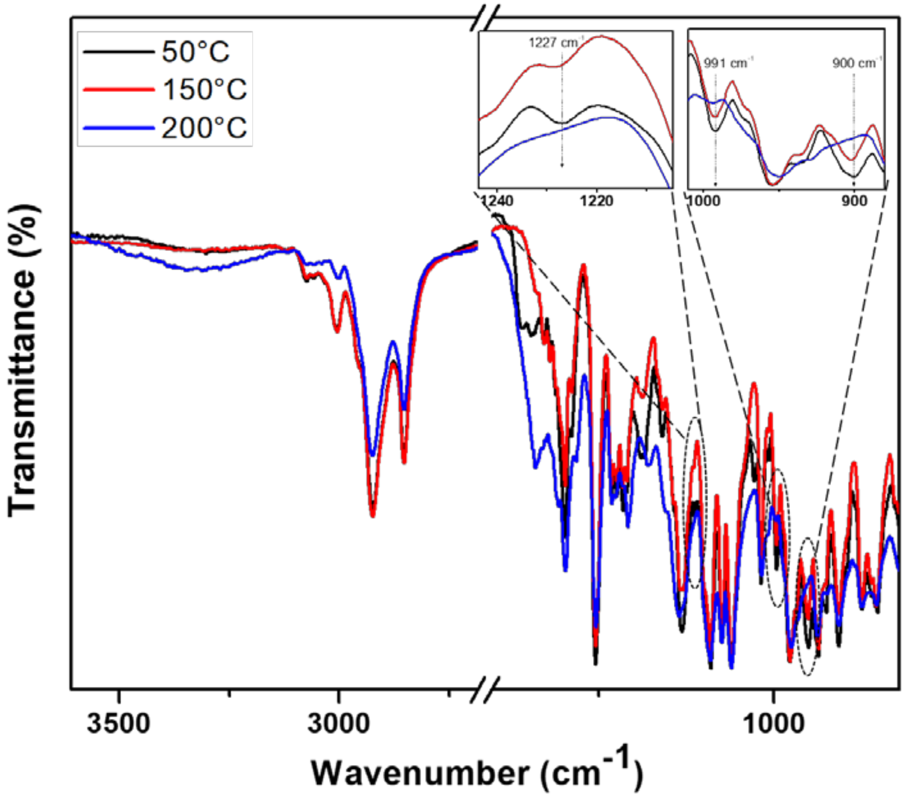

Figure S6. Normalized FTIR spectra for EP_1T_3 heated at 50 °C, 150 °C and 200 °C.

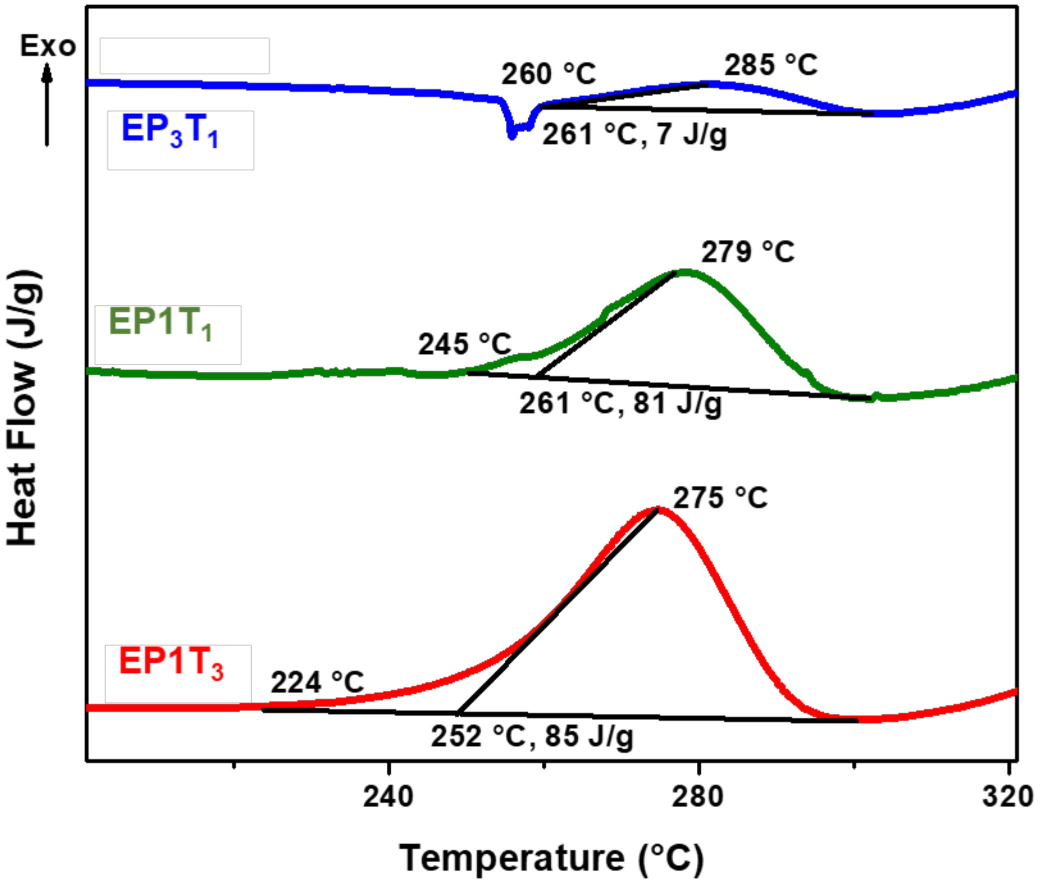

Figure S7. DSC thermograms of EP:C-trisapm monomer blends at different ratio, at a heating rate of 10 °C/min under N_2 atmosphere.

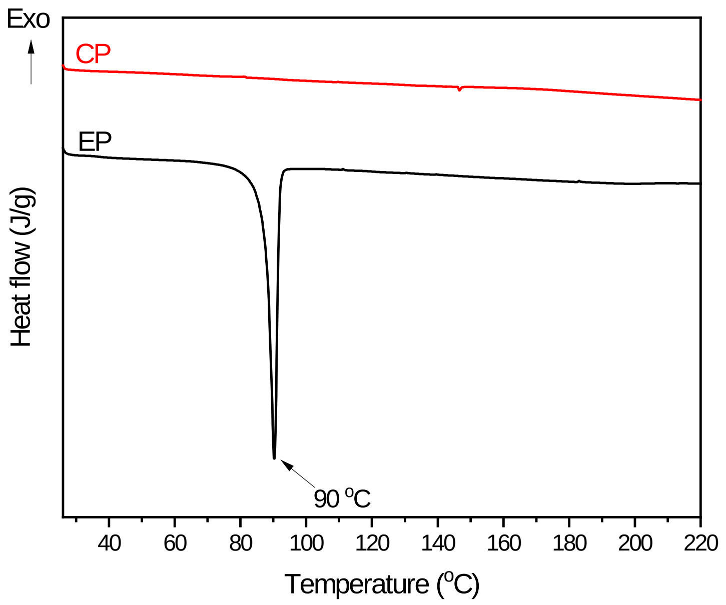

Figure S8. DSC thermograms of EP and CP monomer at a heating rate of 10 °C/min under N_2 atmosphere.

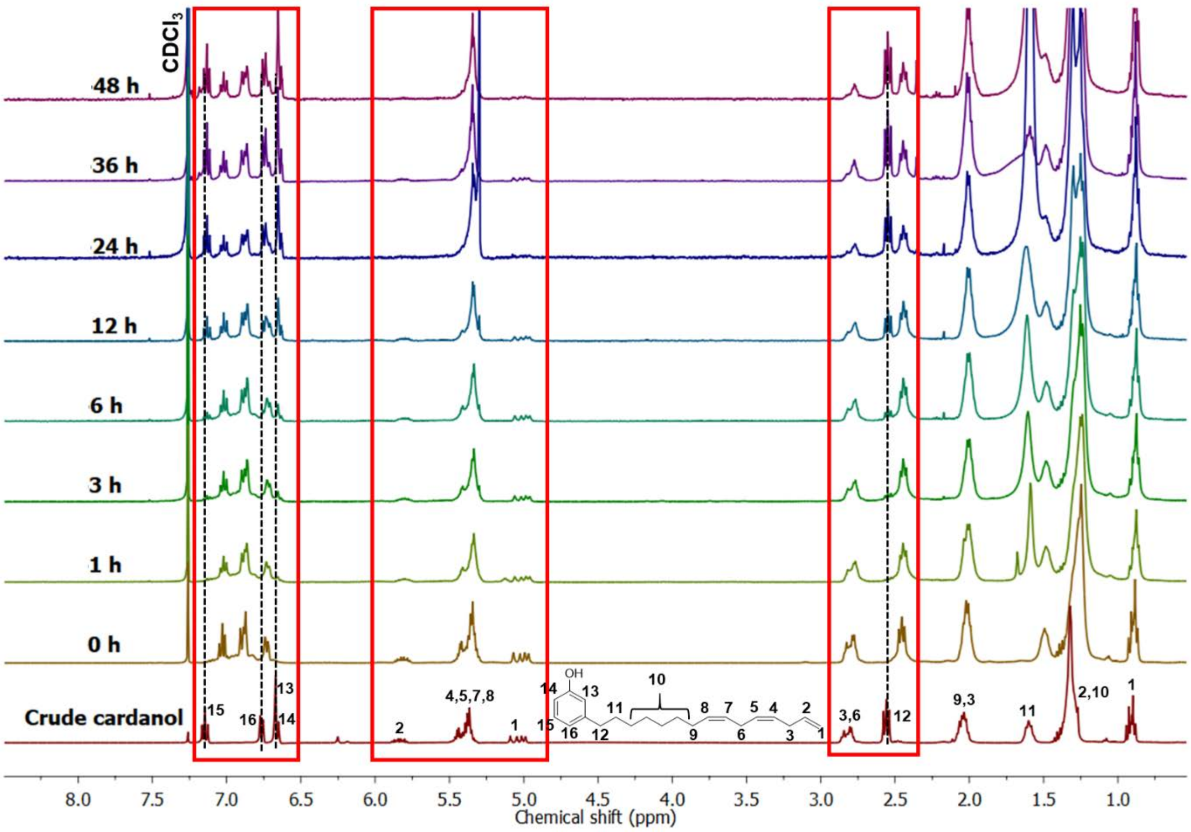

Figure S9. Stacked ^{1}H NMR spectra of cardanol and CP. The monomer CP was heated at 200 °C, under N_2, for different time intervals. Peaks normalized at 2.46 ppm.

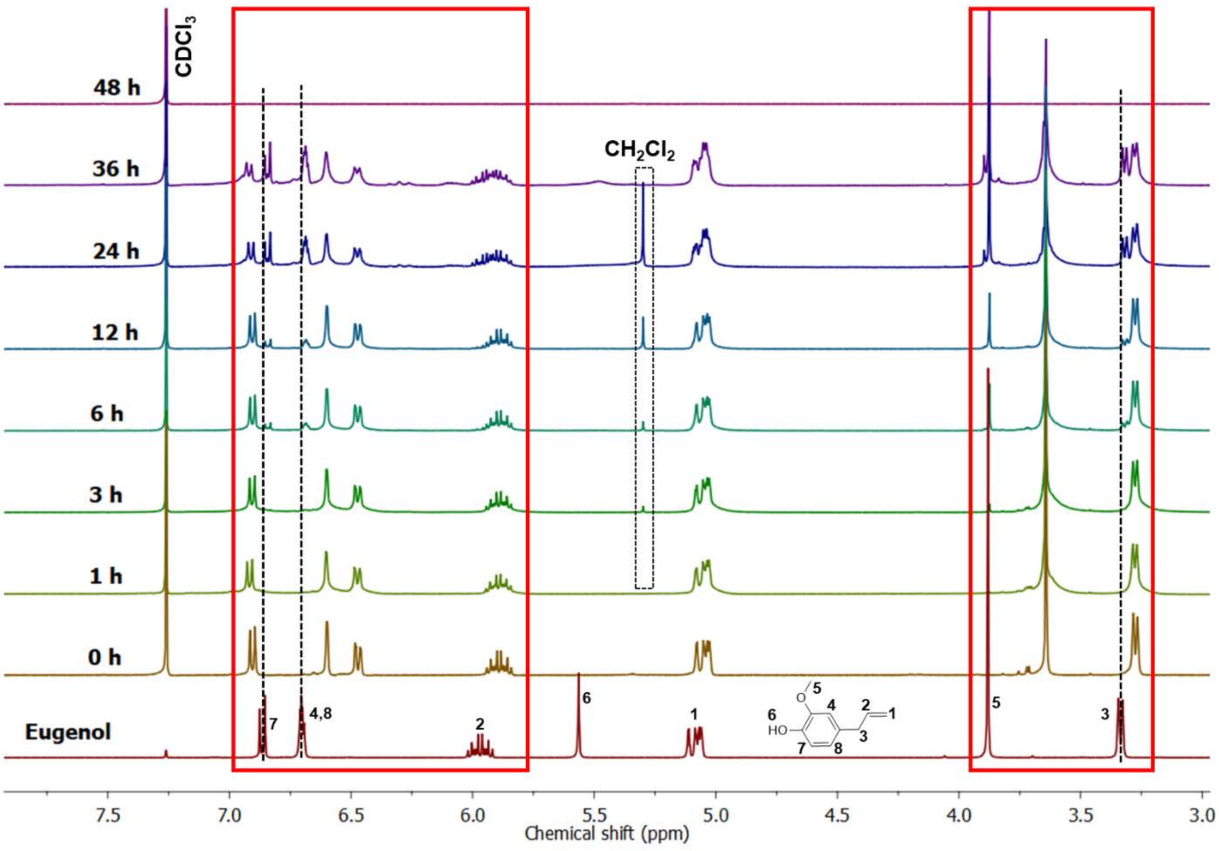

Figure S10. Stacked of ^{1}H NMR spectra of eugenol and EP. EP monomer was heated at 200 °C, under N_2, for different time intervals. Peaks normalized at 3.27 ppm.

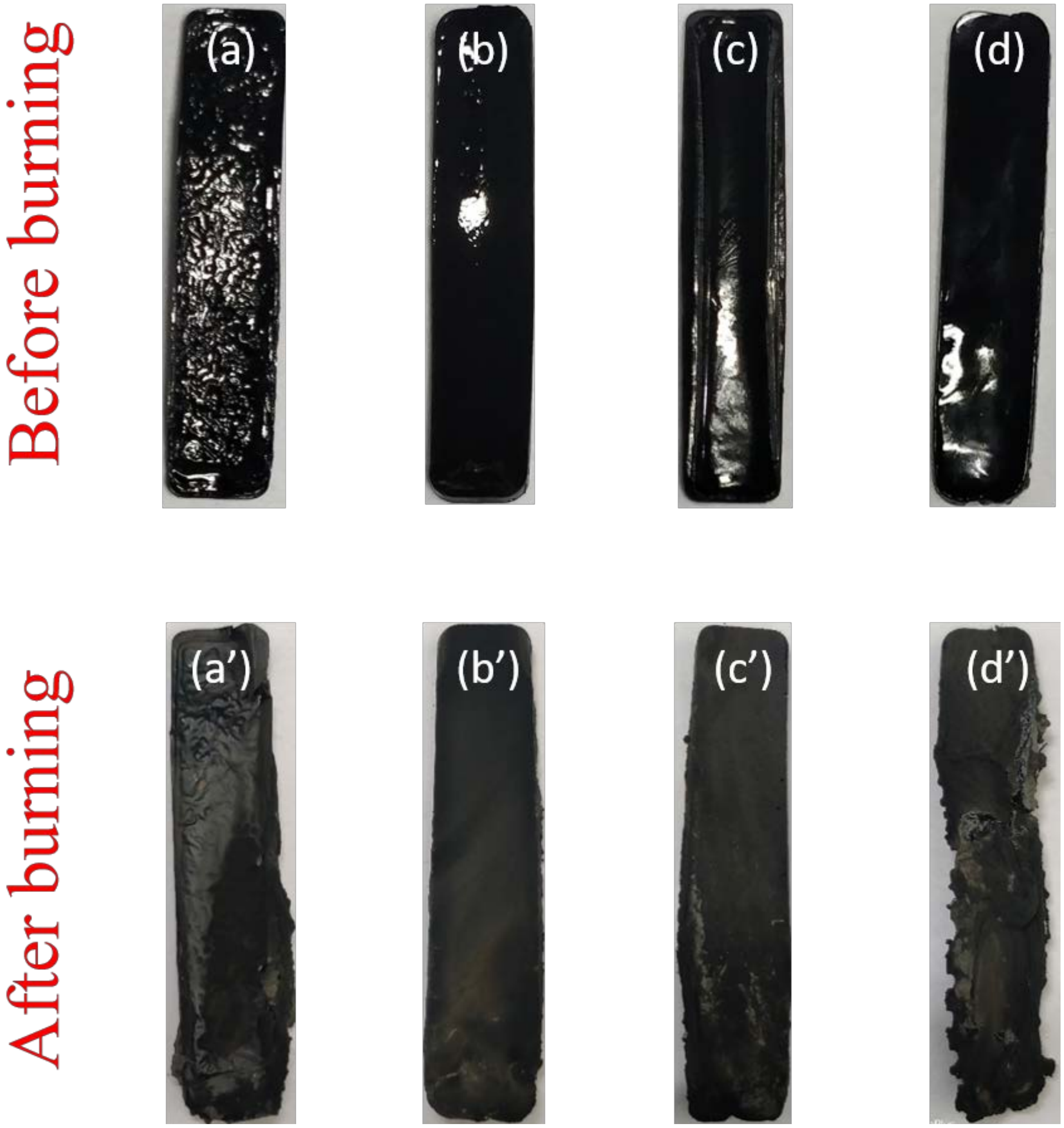

Figure S11. Digital images of the samples which passed the vertical burning test (before and after analysis) (a, a') poly(CP), (b,b') poly(CP_3T_1), (c, c') poly(CP_1T_3), and (d, d') poly(C-trisapm).

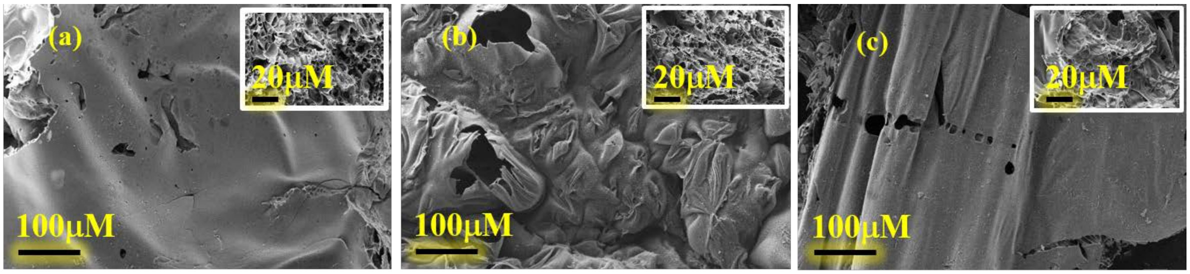

Figure S12. SEM images of external and interior (inset) surfaces of residual char of (a) poly(EP), (b) poly(EP_3T_1), (c) poly(EP_1T_3).

Table S1. Relative atomic % for P and O element before and after burning, determined from EDS analysis.

**Sample**	**Experimental (after burning)**			**Theoretical (before burning)**		
	**%O**	**%P**	**P/O**	**%O**	**%P**	**P/O**
CP	49.2	50.8	1.0	4.9	4.75	1.0
CP_1T_3	59.13	40.87	0.7	4.045	1.19	0.3
CP_3T_1	57.69	42.31	0.7	4.62	3.56	0.8
EP	84.92	15.08	0.2	17.23	8.34	0.5
EP_1T_3	98.08	n.d.	n.d.	7.13	2.09	0.3
EP_3T_1	53.55	46.45	0.9	13.86	6.26	0.5
